# Expanding genomic resources for heritage science: characterization of selected microbial isolates from salt-weathered historic sites

**DOI:** 10.3389/fmicb.2026.1854786

**Published:** 2026-07-02

**Authors:** Lukas Fürnwein, Elias Lehner, Johannes Tichy, Monika Waldherr, Ylenia Vassallo, Beate Sipek, Katja Sterflinger, Guadalupe Piñar, Alexandra B. Graf

**Affiliations:** 1 Department of Applied Life Sciences, Bioengineering, Bioinformatics, Hochschule Campus Wien, Vienna, Austria; 2 Institute for Natural Sciences and Technology in the Art, Academy of Fine Arts Vienna, Vienna, Austria; 3 Department of Medical Biotechnology, University of Siena, Siena, Italy; 4 Institute for Conservation - Restoration, Academy of Fine Arts Vienna, Vienna, Austria

**Keywords:** bioinformatics and computational biology, genome assembly and annotations, genomics, heritage science, microbiology

## Abstract

**Introduction:**

On historic masonry and plaster, moisture-driven salt crystallization cycles impose mechanical stress and create niches for halophilic and halotolerant microbial communities. Halotolerant/halophilic microorganisms isolated from these man-made heritage environments are usually not as well characterized as those isolated from natural environments and represent a genetic and biotechnological potential that has not been thoroughly studied to date.

**Methods:**

This study provides insights into the genomes of five selected halophilic and halotolerant microorganisms isolated from two salt-weathered heritage sites in Austria: the subterranean St. Virgil Chapel beneath St. Stephen’s Cathedral (13th century) and the Charterhouse Mauerbach (14th century). Isolates displaying coloration under 10–20% NaCl were sequenced using Oxford Nanopore long-read technology, yielding complete genomes and plasmids. Functional annotation focused on metabolic, osmoregulatory and pigment biosynthesis pathways to elucidate adaptive strategies underpinning their persistence under extreme salinity.

**Results:**

Comparative genomic analyses revealed variations between the isolated strains with their respective references, as well as species-specific traits that have not been described in detail before and confirmed the presence of robust carotenoid pathways including bacterioruberin synthesis in *Halococcus*, mixed C40/C50 carotenoids in *Nesterenkonia*, and C30/C40 carotenoids in *Halobacillu*s. Furthermore, two isolates, *Marinobacter* sp. 119-V2 and *Modicisalibacter* sp. 110-V3, likely represent novel taxa, indicating that salt-weathered heritage sites represent a specific environmental niche that selects for specific microbial colonizers.

**Discussion:**

By integrating cultivation, phenotypic characterization, and genomic analysis, this work advances beyond descriptive community surveys toward mechanistic understanding of microbial functions relevant to conservation science. Genome-informed insights into pigment biosynthesis and osmotic stress response help predict microbial behavior under environmental or treatment-induced salinity fluctuations, supporting the development of targeted, scientifically grounded preservation approaches for salt-affected cultural heritage.

## 1 Introduction

Microorganisms adapted to highly saline habitats have been extensively characterized in natural environments where salts accumulate, including salt lakes, solar salterns, and salt mines. Halophilic and halotolerant organisms have also been repeatedly identified in built environments, including cultural heritage sites, where salt accumulation likewise occurs. However, in contrast to natural environments, man-made environments remain comparatively underexplored. Consequently, the microorganisms colonizing these habitats, as well as their genetic diversity and biotechnological potential, have been largely overlooked.

Salt accumulation is one of the main problems encountered in the conservation of architectural surfaces. In salt-weathered surfaces, soluble salts are transported by water within the porous network of historic masonry and plaster and further crystallize when the moisture level is reduced. Salt-weathered building materials create a distinct, high-stress micro-environment influenced by alternating desiccation and water ingress cycles, as well as extreme weather events linked to climate change. These conditions result in repeated episodes of high concentration of dissolved ions, low water activity and strong mechanical stress at the stone surface and within its pore network (Tichy et al., 2025). In contrast to many natural hypersaline systems, historic masonry is a rigid, porous mineral substrate, where salts crystallize and dissolve inside narrow pores and at the surface, driven by capillary rise and evaporation (Zhang et al., 2024). This produces strong local gradients of moisture and salt along micrometer-scale pore networks that microorganisms cannot easily escape by movement (Otlewska et al., 2017; Adamiak et al., 2016; Schiro et al., 2012). These cycles impose alternating osmotic conditions, from moderately wet and less saline to extremely saline and desiccating on short time-scales (Delgado et al., 2016). This implies that the microorganisms colonizing these habitats have developed different mechanisms for adapting to constantly changing salinity conditions, which notably differ from the more stable ones found in natural environments.

Specialized halophilic and halotolerant bacteria, archaea, and algae have evolved mechanisms to cope with the resulting osmotic stress. In addition to osmotic stress, salt-weathered facades often combine high irradiance, pronounced desiccation, temperature fluctuations and nutrient limitation. As a result, successful colonizers often show a portfolio of co-selected traits, including carotenoid-based pigmentation (e.g., ruberins) for photoprotection and radical scavenging, robust stress-response systems, thick extracellular polymeric matrices that buffer micro-gradients, and biofilm formation interwoven with salt crystals (Ettenauer et al., 2014; Tichy et al., 2023; Cubero et al., 2025).

In the field of cultural heritage, biofilms associated with salt efflorescence can often be identified by their characteristic pink, orange, or reddish coloration. Carotenoids contribute to membrane stability under high ionic pressure and can improve cell membrane fluidity (Li et al., 2023; Mandelli et al., 2012). Pigments thereby represent an evolutionary advantage for microorganisms living in saline environments. The produced pigments are often very stable and contribute to persistent aesthetic biodeterioration that can mask or damage original finishes (Cojoc et al., 2019). Beyond visual impact, halophilic colonization is part of a complex biodeterioration process that intersects with physical and chemical decay mechanisms. The same traits that enable survival under these conditions, such as pigment production and biofilm formation, are directly linked to visible and material alteration of heritage surfaces, making this a conservation-critical microbial niche rather than a marginal one.

Mitigation measures that alter salt load or ion composition (e.g., clay-based poultices, desalination interventions) also act, unintentionally, as ecological manipulations of the resident microbiota, as shown by long-term monitoring of community shifts during such treatments. Recent work on “pink biofilms” couples chemical analysis with molecular techniques to track community shifts during desalination treatments, showing that changes in salt load and composition can selectively impact bacterial versus archaeal pigment-producers (Tichy et al., 2025). Furthermore, it has been proposed that color signatures (e.g., carotenoids, chlorophylls, scytonemin) could function as non-invasive proxies for community composition, physiology, and environmental stress on stone and other mineral substrates, with clear applications for risk assessment and monitoring of cultural heritage affected by salinity-driven biodeterioration (Villa et al., 2022).

In this context, the present study contributes to the growing genomic reference pool for the analysis of microbiomes in art and cultural heritage by focusing on five microorganisms tolerant to high salt concentrations, isolated from two highly saline heritage sites: the St. Virgil Chapel (13th century), situated below ground, underneath the famous St. Stephen’s cathedral in Vienna, and the Charterhouse Mauerbach (14th century), a historic cloister complex, situated in Mauerbach (Lower Austria). The organisms were selected based on their ability to thrive under hypersaline conditions and in the case of some of them, for their production of visible pigments, two traits of relevance in biodeterioration processes. Furthermore, the selected microorganisms have been frequently identified in several studies performed on geographically distant salt-weathered monuments. The isolates were long-read sequenced with Oxford Nanopore Technologies, and subsequently *de novo* assembled. All isolates could be assembled into full genomes including their plasmids. The genome sequences were annotated and compared to preexisting data with a focus on metabolic, osmoregulation and pigment producing pathways. By generating *de novo* genome assemblies for all five isolates and performing detailed comparative and functional genomic analyses, this work moves beyond descriptive surveys of microbial presence and instead, it addresses the functional potential and adaptive strategies of specific microorganisms well adapted to changing environmental conditions, directly implicated in built cultural heritage deterioration.

The use of *de novo* genome sequencing represents a methodological advance for the study of cultural heritage microbiomes. While amplicon-based approaches have been instrumental in revealing microbial diversity across a wide range of heritage contexts, they provide limited insight into metabolic capabilities, stress responses, and interactions with substrates. Whole genome-based analyses enable the identification of specific pathways and processes that underpin microbial survival and activity in extreme environments. In line with the priorities of systems microbiology, this study combines high-quality genomic data with a clear functional focus, offering insights into the mechanisms that are relevant to understand how microorganisms survive in man-made saline environments that have *hitherto* been relatively unexplored, and which have direct applications for conservation science.

## 2 Materials and methods

### 2.1 Isolation and cultivation of microbial strains

The 5 microorganisms investigated in this study were selected from enrichment liquid cultures, performed as previously described (Piñar et al., 2016), from which over 141 colonies were obtained and 68 pure strains of different morphology and appearance were obtained (data non-published). They were enriched in 10 and 20% NaCl by using two different media: Maintenance Medium (HMM, 10% w/v NaCl) (Spring et al., 1996), and M2 medium (20% w/v NaCl) (Tomlinson and Hochstein, 1976). The cultures were grown in 300-ml Erlenmeyer flasks containing 30 mL of the media mentioned above. They were inoculated with samples of salt-weathered architectural surfaces, obtained from two historic buildings: the chapel of St Virgil (Vienna) and the Charterhouse Mauerbach (Lower Austria), weighing approximately 90–120 mg. To avoid fungal growth, media were supplemented with 50 µg/ml^-1^ cycloheximide (Sigma). Flasks were incubated aerobically at room temperature (RT) (20–22 °C) by shaking at 180 rpm (Thermo Scientific Equipment MaxQ 8,000, Germany) for a period of 4–5 weeks. Over the total period of incubation, weekly aliquots of 100 μL were serial-diluted and plated onto the same solid media (without cycloheximide) and incubated aerobically at RT for 2–10 weeks, depending on the growth of the microorganisms. The colony morphology was examined on an Olympus SZX9 phase contrast microscope. Colonies showing different morphology and pigmentation were transferred to new culture plates to obtain pure cultures. Pure strains were further cultivated in fresh media until exponential growth occurred, to be stored finally in 70% (v/v) glycerol at −80 °C for preservation.

### 2.2 DNA extraction, 16S rRNA gene PCR amplification and sequencing

To identify the selected microorganisms, genomic DNA was extracted according to the protocol provided by (Wilson, 2001). The DNA yields were quantified using a Qubit 2.0 fluorometer (Thermo Fisher Scientific, Waltham, MA, USA) with the Qubit dsDNA HS Assay Kit. PCR was performed in a BioRad C1000 Thermal Cycler. The Promega (Madison, Wisconsin, USA) premixed PCR Mastermix, 2X [50 units/mL of Taq DNA polymerase supplied in a proprietary reaction buffer (pH 8.5), 400 and μM dATP, 400 and μM dGTP, 400 and μM dCTP, 400 and μM dTTP, and 3-mM MgCl^2^] was diluted to 1X and 0.5 pmol/μL of each primer (stock: 50 pmol/mL) was added. Between 10 ng and 20 ng of template DNA was added in a total reaction volume of 50 μL.

For sequencing of the bacterial isolates, 16S rDNA fragments were amplified using the forward primer 27F and the reverse primer 1492R (Stackebrandt and Goodfellow, 1991). The thermocycling program used was as follows: 5 min at 95 °C, followed by 30 cycles consisting of [95 °C, 1 min; 55 °C, 1 min and 72 °C, 2 min] followed by a final extension step for 5 min at 72 °C. For sequencing of the archaeal isolate, 16S rDNA fragments were amplified using the forward primer ARC344 and the reverse primer ARC915 (Raskin et al., 1994). The following thermocycling program was used: 5 min at 95 °C, followed by 40 cycles consisting of [95 °C, 1 min; 60 °C, 1 min and 72 °C, 1 min] with a final extension step for 5 min at 72 °C.

The resulting PCR products were purified using the QIAquick PCR Purification Kit (Qiagen, Hilden, Germany) and analyzed by electrophoresis on a 2% (w/v) agarose gel. Sanger sequencing of the PCR products was performed through Eurofins Genomics (Vienna, Austria) and the resulting nucleotide sequences were compared to those in the online databases provided by the National Center for Biotechnology Information (NCBI) using the BLAST search tool. The generated 16S rDNA sequences from the selected strains have been deposited in the NCBI database under the accession numbers shown in Table 1.

**Table 1:** Phylogenetic classification of the four isolated bacterial- and one archaeal- strain from two salt-weathered buildings.

[NaCl] (%)	Strain No.	Strain name	Phenotype (colour)	Closest phylogenetic relatives [EMBL accession numbers]	Similarity (%)	Access. Nr.
10%	8	41-M4	Pale pink	*Halobacillus amylolyticus* strain SSHM10-5 isolated from saltern [CP095075.1]	99.59	PV467619
	11	110-V3	Beige to yellowish	*Halomonas muralis* isolate 0612R7A-3 isolated from salt-weathered historical buildings with mural paintings [HG515395.1] PUBMED 25084531	99.23	PV467622
	13	118-M6	Yellow	*Nesterenkonia xinjiangensis* strain TZNB-2 [MF170854.1] isolated from the surface of stalactite.	99.85	PV467624
	14	119-V2	Bright pale pink	*Marinobacter iranensis* strain 71-I isolated from Inche broun hypersaline lake in Iran (Rafieyan et al. DOI: 10.1099/ijsem.0.006083) [MK101100.2]	99.72	PV467625
20%	21	11-V1	Dark pink	*Halococcus salifodinae* strain YPL10 [KX898185.1]	99.45	PV467631

V1, V2, V3: strains isolated from the St. Virgil Chapel from the sampling points V1, V2 and V3, respectively (Tichy et al., 2023). M4, M6: strains isolated from the Charterhouse Mauerbach from the two sampling points M4 (room 1) and M6 (room 2), respectively (Tichy et al., 2023).

### 2.3 DNA extraction, library preparation and nanopore sequencing

The selected isolates were then further cultivated, with the gram-negative bacteria *Halomonas* sp. and *Marinobacter* sp., as well as gram-positive bacteria *Nesterenkonia* sp. and *Halobacillus* sp., being cultivated in Tryptic Soy Broth (Merck, cat. no. 1054590500) supplemented with 10% NaCl (w/v) and 2% MgSO4 (w/v). The archaeal isolate, *Halococcus* sp., was cultivated in M2 medium (20% w/v NaCl). Afterwards the collected biomass was pelleted and washed twice with saline solution (10% NaCl [w/v]).

The DNA of all selected strains was extracted using the Purgene Cell Kit. The protocol for *Halomonas* sp. and *Marinobacter* sp. was performed according to the section on gram-negative bacteria (Qiagen cat.no 158043, HB-0326-004_HB_Puregene_DNA_0122_WW), with the isopropanol including step (No 10.) being extended overnight at 4 °C. The DNA hydration (step. No 17) was done in TE (10 mM Tris–HCl, 1 mM EDTA, pH 8.0) according to the recommendations of ONT. *Nesterenkonia* sp.*, Halobacillus* sp. and the archaeal *Halococcus* sp. were extracted according to the manufacturer’s recommendations for gram-positive bacteria, with the TE-buffer (100 mM Tris–HCl, 50 mM EDTA, pH 7.7) being used for the cell resuspension and modifications in the enzyme solutions added during step 5.: 50 μL lysozyme (50 mg/mL) + 1 μL lytic enzyme solution (Qiagen, cat. no. 158928) and during step 9.: 10 μL Proteinase K (20 mg/mL).

Sequencing was performed using the Ligation Sequencing Kit V14 (SQK-LSK114) according to the manufacturers’ instructions (Oxford Nanopore Technologies, Oxford, UK; New England Biolabs, Ipswich, MA, USA). Briefly, genomic DNA was subjected to repair and end-preparation using the NEBNext FFPE DNA Repair Mix (NEB, cat. no. M6630) and the NEBNext Ultra II End Repair/dA-Tailing Module (NEB, cat. no. E7546), following the manufacturer’s protocols. Subsequently, DNA was purified and concentrated using AMPure XP beads (provided within the kit), including wash steps with 80% (v/v) ethanol. Adapter ligation was carried out using the NEBNext Quick Ligation Module (NEB, cat. no. E6056), followed by an additional purification step employing the Long Fragment Buffer supplied with the kit. Finally, 50 fmol of the prepared library were loaded onto an R10.4.1 flow cell and sequenced for 48 h.

### 2.4 Data processing and bioinformatic analyses

Raw POD5 files were basecalled using the Dorado v0.7.4 (Oxford Nanopore Technologies, 2024) employing the super accuracy (SUP) model. Read quality was assessed with FastQC (v0.12.1) (Andrews et al., 2010) and NanoStat (v1.6.0) (De Coster et al., 2018) before being filtered with Filtlong (v0.2.1) (Wick, 2018) with a minimum length threshold of 1,000–2,000 bp, retaining the top 90% of reads based on quality scores, and trimming the dataset to a total of 1 Gbp.

Genome assemblies were generated using Flye (v2.9.5-b1801) (Kolmogorov et al., 2019) and subsequently polished with Medaka (v2.0.0) (Oxford Nanopore Technologies, 2020). Assembly completeness and contamination were evaluated using CheckM (v1.2.3) (Parks et al., 2015).

Functional annotation was performed with the EBI Metagenomics pipeline mettannotator (v1.4.0). Taxonomic classification of the final assemblies was conducted using the GTDB-Tk (Chaumeil et al., 2020), initially applying the *classify_wf* workflow. For genomes that did not achieve species-level resolution, phylogenetic placement was refined using the *de_novo_wf* workflow. *De novo* phylogenetic trees of *Marinobacter* and *Modicisalibacter* were rooted with members of the *Alphaproteobacteria* as an outgroup. The GTDB reference tree was subsequently pruned based on GTDB taxonomy to retain only representatives of the genera *Marinobacter* and *Modicisalibacter*. Whole-genome average nucleotide identity (ANI) (Jain et al., 2018) was calculated for each newly assembled isolate and its reference, in cases where the reference species/strain was not clearly identifiable, the ANI was calculated for the most likely candidates, confirming the results of the phylogenetic tree. Extracellular protease analysis was conducted using DeeplLocPro (1.0) (Moreno et al., 2024), HotPep (2020-11-18) (Busk, 2020) on the MEROPS database (12.4) (Rawlings et al., 2018) and mettannotators dbcan output. Comparative analyses between newly assembled genomes and reference annotations were performed using CD-HIT (v4.8.1) (Fu et al., 2012) with the parameter -c 0.9 and BLASTp (v2.16.0+) (Altschul et al., 1990), both run with standard parameters. Synteny visualization was conducted with JupiterPlot (v1.1) (Chu, 2018), applying maximum gap thresholds of 5,000 and 50,000 bp, as well as a minimum size of a contiguous region of 25,000 bp and the minimap2 parameter -x asm20 to generate alignments, where asm20 corresponds to the following parameters: -k19 -w10 -U50,500 --rmq -r1k,100 k -g10k -A1 -B4 -O6,26 -E2,1 -s200 -z200 -N50.

## 3 Results

The microorganisms selected for this study were isolated as part of a wider study dedicated to the isolation and identification of halotolerant and halophilic microorganisms from architectural structures damaged by salt and displaying discolorations (Supplementary Figure S1). Isolations were carried out at salt concentrations ranging from 3–20%, and around 200 microorganisms were cultivated. The main selection criteria to use these five specific strains for subsequent whole-genome sequencing were their high salt tolerance under saline conditions (10–20%), similar to those found in the previously investigated historic buildings, displaying areas with salt efflorescence (Tichy et al., 2023), and their capability of pigment production. These two characteristics are fundamental for their use as model organisms for future investigations in salt-weathered surfaces. Regarding salt tolerance, the selected bacteria were all isolated at concentrations of 10% NaCl, while the archaeal strain was isolated at a salt concentration of 20% NaCl. Regarding pigmentation, the bacterial strains exhibited colors ranging from yellowish beige to yellow and light pink, while the archaeon displayed a very intense dark pink color. However, in addition to these two key criteria, other important considerations were also taken into account for their selection as model organisms: (a) their relative abundance in the core microbiome previously detected via 16S rDNA data; (b) their frequent identification in studies performed on geographically distant salt-weathered monuments; (c) the interest to use their genomes as reference genomes for future data-mining and transcriptome studies.

Taxonomic 16S rDNA sequence analysis (Table 1) showed that the 5 selected strains were affiliated with 4 different bacterial genera, within 3 phyla: the *Bacillota*, the *Actynomicetota,* and the *Pseudomonadota.* The strain no. 8 (41-M4), isolated from a sample from the Charterhouse Mauerbach, was assigned to the genus *Halobacillus*, phylum *Bacillota*. Strain no. 13 (118-M6), which was also isolated from the Charterhouse Mauerbach, was assigned the genus *Nesterenkonia* from the phylum *Actynomicetota*. Finally, the phylum *Pseudomonadota* was represented by the genus *Marinobacter* (strain no. 14, 119-V2) and the genus *Halomonas* (strain no. 11, 110-V3), both isolated from the St. Virgil Chapel. In addition, another strain from the St. Virgil Chapel (no. 21, 11-V1) was assigned the archaeal species *Halococcus salifodinae* of the order *Halobacteriales*, phylum *Methanobacteriota* (Table 1).

### 3.1 Sequencing, assembly and annotation

Raw Oxford Nanopore sequenced reads were characterized by a high sequencing depth (number of reads, total bases), but relatively short read lengths and lower quality scores (Supplementary Table S1). Through a rigorous filtering process both mean and median read length as well as quality scores could be improved, while still retaining enough sequencing depth for high quality assemblies (Supplementary Table S1). This trend is consistent across all samples, indicating that filtering effectively enriches for longer, higher-quality reads. All *de novo* assemblies resulted in a large circular contig representing the main chromosome of the species. The reads of *Halococcus* sp. 11-V1 contained additionally four smaller circular contigs. The completeness of the 5 newly assembled genomes ranged from 98.57 to 100% and the contamination estimate from 0.07 to 1%. These assembled genomes were then compared with their closest reference determined through taxonomic classification and average nucleotide identity (ANI), with organisms belonging to the same species typically showing ≥ 95% ANI among themselves (Konstantinidis and Tiedje, 2005).

#### 3.1.1 *Halococcus* sp. strain 11-V1

*De novo* assembly of the filtered reads of *Halococcus* sp. strain 11-V1 resulted in one large circular contig representing the main chromosome with a length of 3,447,141 bp and four smaller circular contigs with lengths of 246,799 bp; 139,685 bp; 59,155 bp and 51,153 bp, with high completeness (98.57%) and low contamination (0.99%) over the whole assembly (Table 2; Supplementary Table S1). Annotation with the mettannotator pipeline predicted 4,008 protein coding genes, 3 rRNA genes and 48 tRNA genes (Table 2; Supplementary Figure S2). The genome was classified as *Halococcus salifodinae*, showing the highest similarity to the reference genome GCF_052043865.1 with an ANI of 98.65%.

**Table 2:** Assembly and annotation statistics of the five isolated microorganisms.

Isolate	*Halococcus* sp. strain 11-V1	*Nesterenkonia* sp. strain 118-M6	*Halobacillus* sp. strain 41-M4	*Marinobacter* sp. strain 119-V2	*Modicisalibacter* sp. strain 110-V3
Contigs	5	1	1	1	1
Coverage	255	138	232	111	258
Total genome size (Mb)	3.9	3.67	4.22	4.49	3.88
Completeness %	98.57	99.28	99.33	100	99.93
Contamination %	0.99	0.46	1	0.07	0.54
GC %	63.45	68.67	40.9	57.19	59.71
Genes (CDS)	4,008	3,377	4,363	4,151	3,708
rRNA	3	6	19	9	12
tRNA	48	47	69	51	64

The reference genome of *Halococcus salifodinae* also consists of five contigs with the main chromosome being 295,695 bp smaller than the newly assembled strain, and the four contigs being considerably larger (with 401,256 bp; 351,008 bp; 255,175 bp and 87,840 bp respectively). The reference had lower completeness (94.49%) and higher contamination values (18.82%) compared to the strain 11-V1 assembly (Supplementary Table S1). A synteny analysis revealed a high level of agreement except for the integration of parts of the contig NZJBQEMN010000002.1 into the main chromosome, with further divergences between the genomes in the region around the integration, as well as low synteny between the other smaller contigs, which may represent plasmids (Supplementary Figure S3). For *Halococcus* sp. strain 11-V1, a lower number of protein coding (−225) and tRNA genes (−3) were predicted in comparison to the reference. Further analysis, comparing not only gene sequences, but encoded protein functions, revealed 15 encoded protein products to be only present in strain 11-V1 (9 on the main chromosome, 3 on contig2, 3 on contig3), and 39 that were missing from the new strain. The functions of the genes only found in strain 11-V1 belong to the Cluster of Orthologous Genes (COG) category “Cell wall/membrane/envelope biogenesis” and “Replication, recombination and repair”. Genes that were not found in the new strain belonged mainly to the categories “Energy production and conversion” and “Carbohydrate transport and metabolism” (Supplementary Table S3).

#### 3.1.2 *Nesterenkonia* sp. strain 118-M6

Filtered reads of *Nesterenkonia* sp. strain 118-M6 were *de novo* assembled into a single circular contig with a length of 3,679,806 bp and a completeness of 99.28% and a contamination estimate of 0.46%. Gene prediction resulted in 3,377 protein coding genes, 6 rRNA and 47 tRNA genes (Table 2; Supplementary Figure S3). The genome was classified as *Nesterenkonia xinjiangensis*, showing the highest similarity values to the reference genome GCF_013410745.1 with an ANI of 97.61%.

The genome of the reference strain of *N. xinjiangensis* is smaller (−110,436 bp), with lower completeness (94.61%) and higher contamination values (11.51%) (Supplementary Table S1). The synteny analysis showed a high level of agreement between the two genomes (Supplementary Figure S5). The number of predicted coding genes was higher (+178) in *Nesterenkonia* sp. 118-M6, whereas the numbers for rRNA and tRNA genes agreed with the reference. A comparison between the gene sequences showed that 125 genes did not have an alignment based or functional representation in the reference and were therefore considered to be specific to strain 118-M6. Of the 125 genes 16 had no assigned COG category and 19 were assigned the category “Function unknown.” Beside these, the three most abundant categories were “Replication, recombination and repair”, “Inorganic ion transport and metabolism” and “Amino acid transport and metabolism”. On the other hand, 81 genes were specific to the reference, with the majority belonging also to the category “Replication, recombination and repair”, and “Transcription”. Further 18 were assigned the “Function unknown” category, and 12 had no COG assignment (Supplementary Table S4).

#### 3.1.3 *Halobacillus* sp. strain 41-M4

*De novo* assembly of *Halobacillus* sp. strain 41-M4 resulted in a single circular contig with a length of 4,223,345 bp, 99.33% completeness and a contamination level of 1.00%. Gene prediction resulted in 4,363 protein coding sequences as well as 19 rRNA genes and 69 tRNA genes (Table 2; Supplementary Figure S6). The genome was classified as *Halobacillus amylolyticus*, showing highest similarity to reference genome GCF_022921115.1 with an ANI of 98.72%.

In comparison, the genome of the established reference, *H. amylolyticus,* consists of a main chromosome with a length of 4,154,097 bp as well as a smaller contig with a length of 145,254 bp. The overall quality of the reference genome was lower (completeness 82.73%, contamination 12.66) than the new strain 41-M4 assembly (Supplementary Table S1). For *Halobacillus* sp. 41-M4 only one circular chromosome was assembled, with no indication of another contig being present in the reads of the isolate. Apart from a few areas, the two genomes have a high level of synteny (Supplementary Figure S7). In comparison strain 41-M4 contained a lower number of predicted protein coding genes (− 19) and fewer rRNA (−5) and tRNA (−1) genes. The genome of *Halobacillus* sp. 41-M4 contained 226 predicted protein coding genes that did not have a correspondence in the reference. Of these 30 had no associated COG category and 47 proteins were assigned the “Function unknown” category. Otherwise, the most frequent categories were “Carbohydrate transport and metabolism”, “Transcription” and “Cell wall/membrane/envelope/biogenesis.” On the other hand, 167 encoded proteins were only present in the reference, of which 30 had no assigned category and 38 the “Function unknown” category. The remaining sequences fell into the categories “Replication, recombination and repair”, “Carbohydrate transport and metabolism” and “Cell wall/membrane/envelope biogenesis” (Supplementary Table S5).

#### 3.1.4 *Marinobacter* sp. strain 119-V2

**Figure 1:**
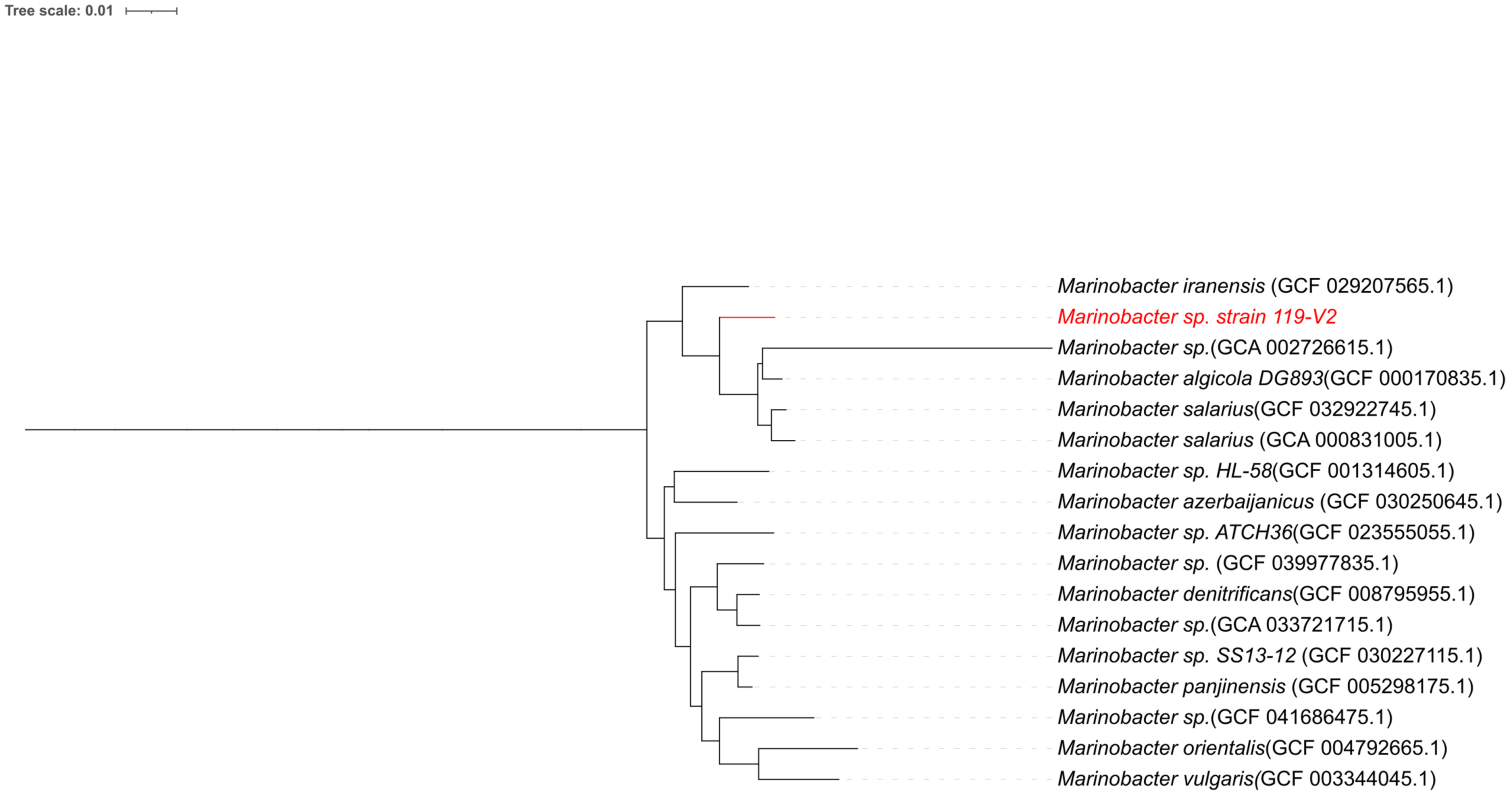
Whole genome phylogenetic tree of the *Marinobacter* genus. The tree is rooted with Alphaproteobacteria as an outgroup. The tree was pruned to only include close relatives within the *Marinobacter* genus. *Marinobacter* sp. strain 119-V2 is shown in red.

A single circular contig with a length of 4,490,321 bp resulted from the *de novo* assembly of *Marinobacter* sp. strain 119-V2. With a completeness of 100% and a contamination of only 0.07% this assembly had the highest overall quality of the 5 isolates. Gene prediction resulted in 4,151 protein coding sequences, 9 rRNA genes and 51 tRNA genes (Table 2; Supplementary Figure S8). Taxonomic assignment at species level using the GTDB-Tk *classify_wf* workflow was unsuccessful. Average nucleotide identity analysis against all available *Marinobacter* reference genomes revealed a maximum ANI of 86.5% with *M. salarius* (GCF_002116735), 86.1% with *M. algicola* (GCF_000170835) and 85% with *M. iranensis* (GCF_029207565), all well below the species delineation threshold of 95%. Whole genome phylogenetic analysis placed the assembled *Marinobacter* sp. 119-V2 strain as a distinct lineage within the genus *Marinobacter*, supporting its designation as a novel species (Figure 1). The placement of *M. salarius* and *M. algicola* its closest relatives, is in line with the ANI results.

**Figure 2:**
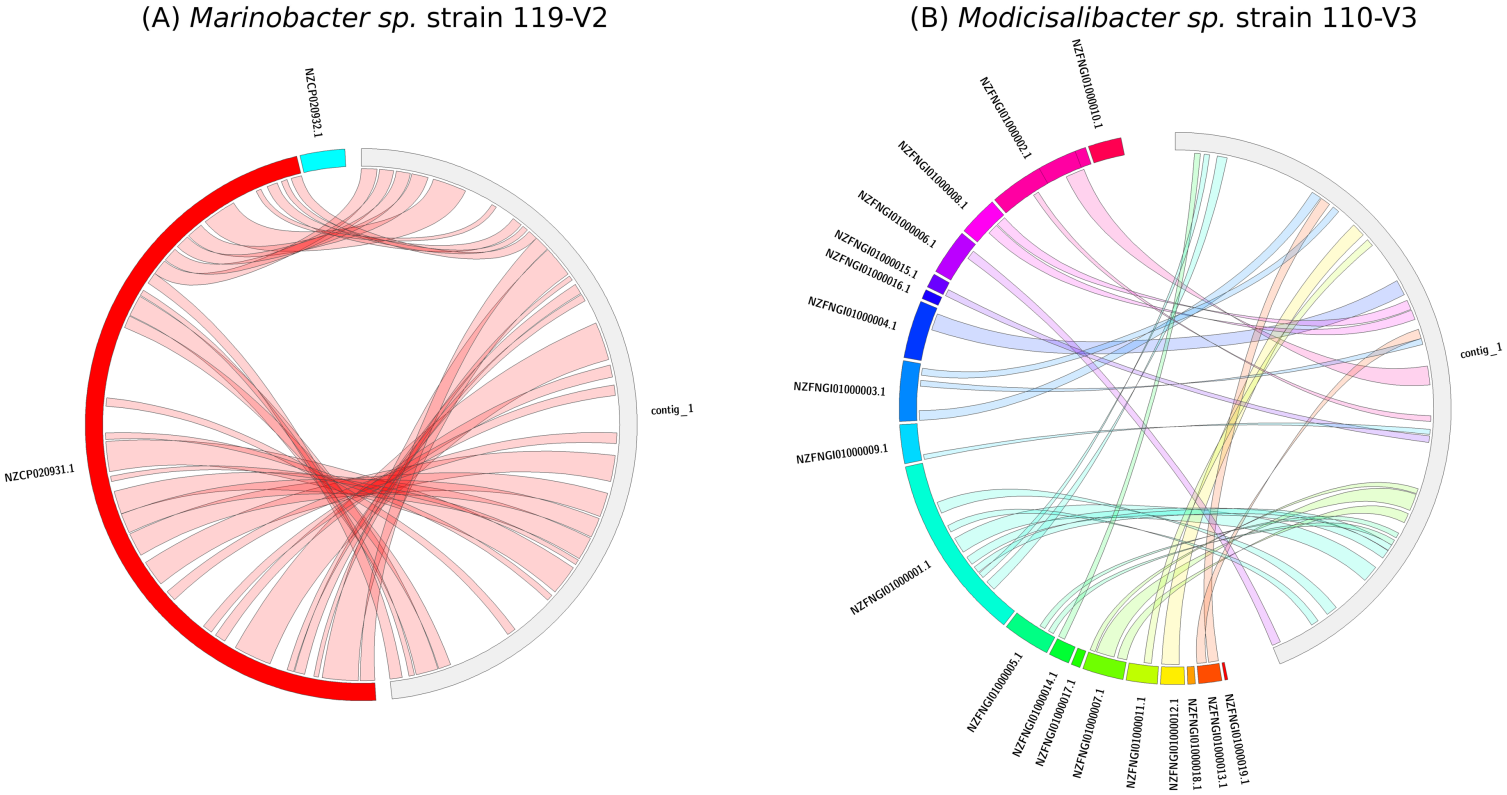
Synteny analysis between **(A)** *Marinobacter* sp. 119-V2 and *Marinobacter salarius* (GCF_002116735); **(B)** *Modicisalibacter* sp. 110-V3 and *Modicisalibacter muralis* (GCF_900102945).

**Figure 3:**
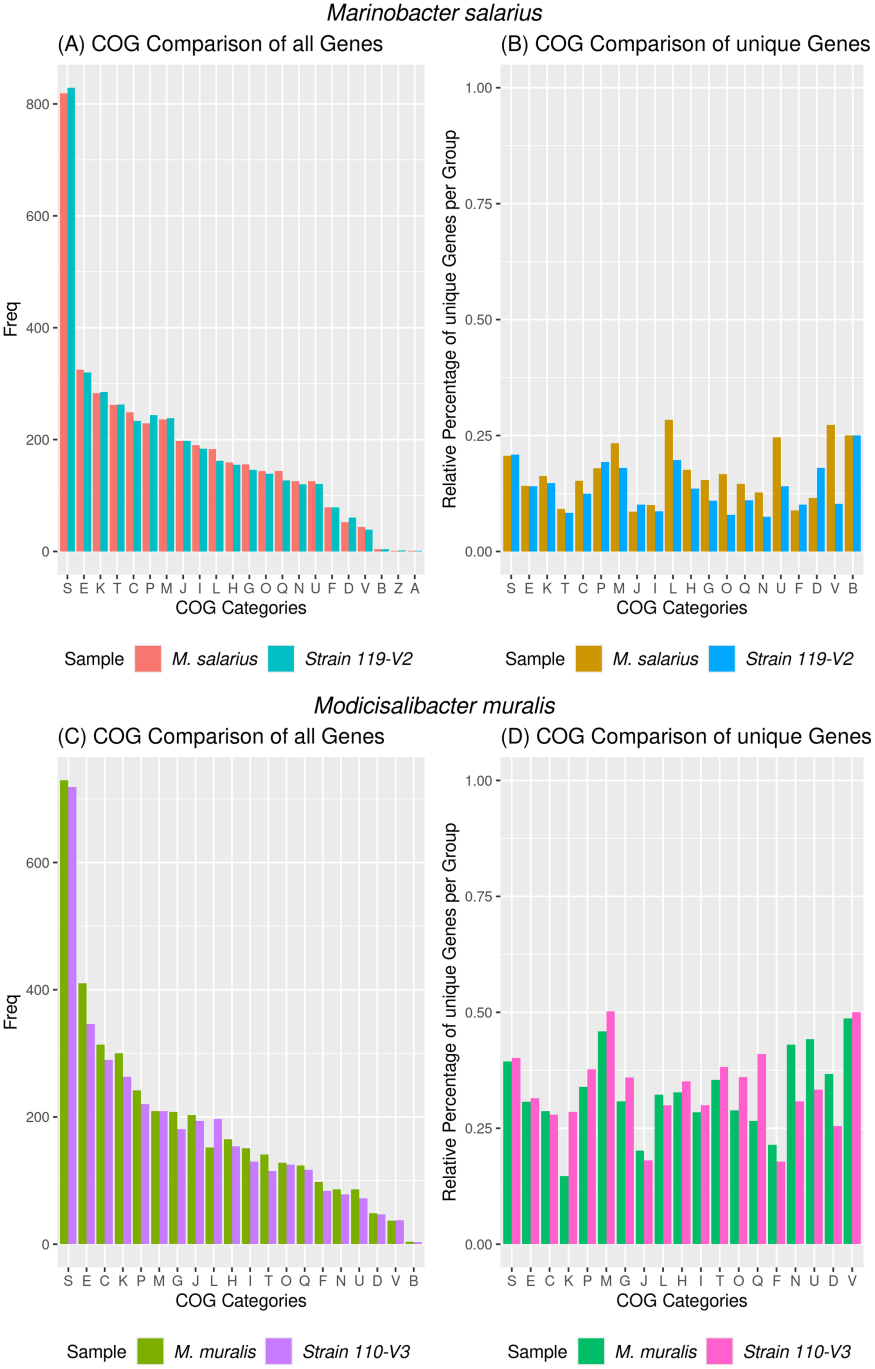
Comparison of genes associated with COG categories between *Marinobacter* sp. 119-V2 and *Marinobacter salarius* (GCF_002116735). **(A)** Comparison of COG categories of all annotated genes; **(B)** comparison of COG categories of non-homologous genes, normalized by the gene count within each category. Comparison of genes associated with COG categories between *Modicisalibacter* sp. 110-V3 and *Modicisalibacter muralis* (GCF_900102945). **(C)** comparison of COG categories of all annotated genes; **(D)** comparison of COG categories of non-homologous genes, normalized by the gene count within each category.

Due to its higher ANI value *M. salarius* was used as a reference for further functional comparison. Its genome consists of two contigs: the main chromosome with a length of 4,386,892 bp and a contig/plasmid with a length of 243,268 bp (completeness 99.98%, contamination 1.04%) (Supplementary Table S1). Analysis showed that the plasmid was missing from *Marinobacter* sp. 119-V2, as the sequence was neither integrated into the main chromosome, nor could it be detected in the raw reads. Synteny analysis showed conserved blocks, separated by regions with lower similarity, which contained homologous genes with reduced similarity values, as well as genes only predicted in one of the two genomes (Figure 2A). *Marinobacter* sp. strain 119-V2 had fewer protein coding genes (−132), but one more tRNA gene, and the same number of rRNA genes. A comparison of COG categories showed that the new isolate had a higher gene count only in the categories “Inorganic ion transport and metabolism” (P) and “Cell cycle control, cell division, chromosome partitioning” (D), whereas the reference had a higher gene count in several metabolism related categories, namely “Energy production and conversion”, “Carbohydrate transport and metabolism”, and “Secondary metabolites biosynthesis …” (C, G, Q), in addition to “Replication, recombination and repair (L) (Figure 3A). A more detailed analysis showed 589 specific protein coding genes in strain 119-V2, of which 40 had no assigned COG category and 173 were assigned the “Function unknown” category (S). The reference contained 733 specific proteins, of which 99 had no category assigned and 169 the category “Function unknown” (S). In this organism specific set, the categories “Translation ribosomal structure and biogenesis” (J) and “Nucleotide transport and metabolism” (F) were overrepresented in *Marinobacter* v8 119-V2, whereas categories related to cellular processes and signaling, most prominent “Cell wall/membrane/envelop biogenesis” (M), “Posttranslational modification, protein turnover…” (O), “Intracellular trafficking, secretion…” (U) and “Defense mechanisms” (V), as well as “Replication, recombination and repair (L) and “Transcription” (K) (Figure 3B).

#### 3.1.5 *Modicisalibacter* sp. strain 110-V3

**Figure 4:**
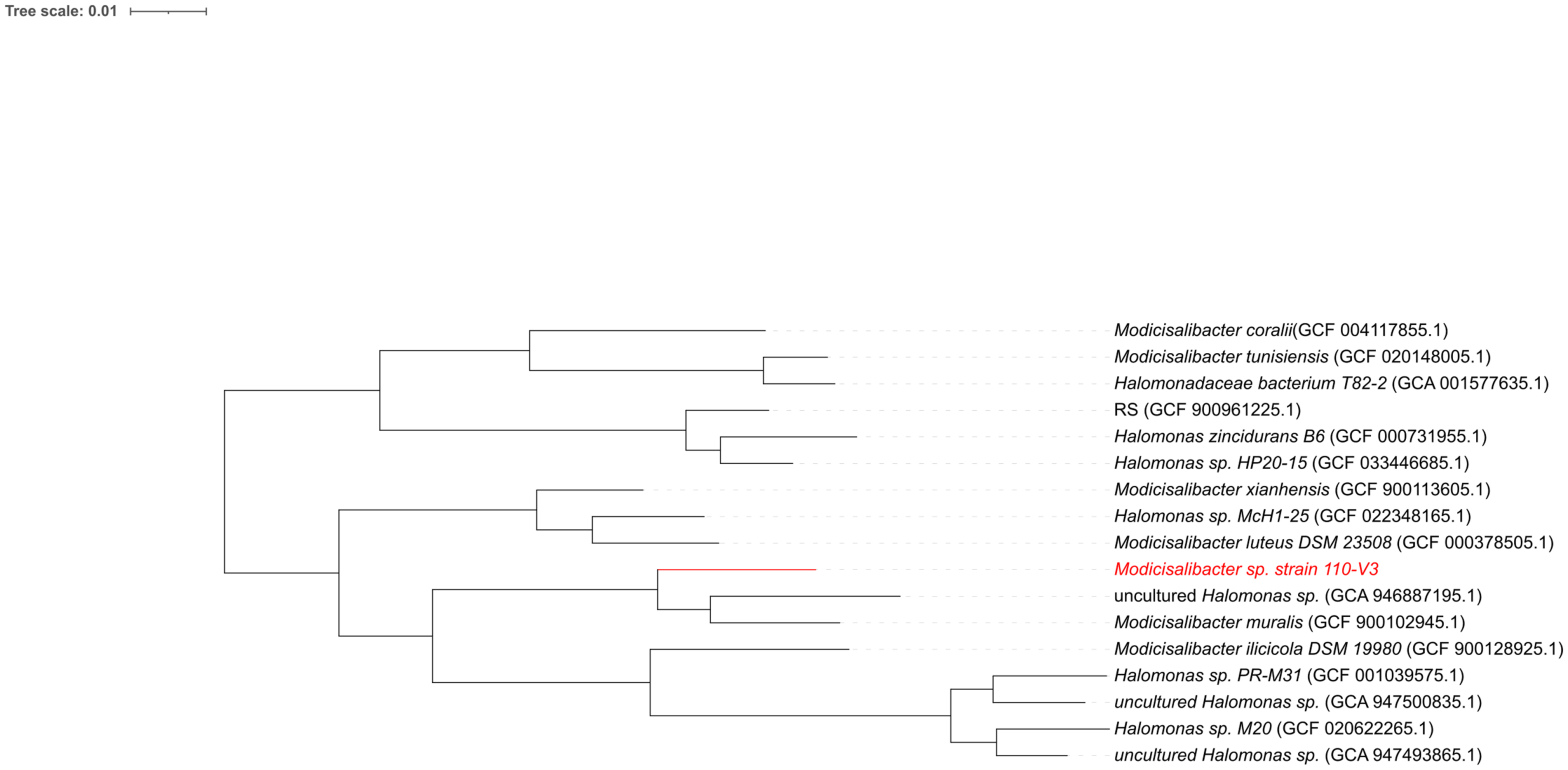
Whole genome phylogenetic tree of the *Modicisalibacter* genus. The tree is rooted with Alphaproteobacteria as an outgroup. The tree was pruned to only include the *Modicisalibacter* genus. *Modicisalibacter* sp. strain 110-V3 is shown in red.

*De novo* assembly resulted in a single contig with a length of 3,883,105 bp, a completeness of 99.93% and only 0.54% contamination. Genome annotation predicted 3,708 coding sequences, 12 rRNA genes and 64 tRNA genes (Table 2; Supplementary Figure S9). Similarly to the *Marinobacter* isolate, taxonomic classification with the GTDB-Tk *classify_wf* workflow did not result in an assignment. Average nucleotide identity analysis against all available *Modicisalibacter* reference genomes revealed a maximum ANI of 85% with *M. muralis* (GCF_900102945) followed by an ANI of 80% with *M. zincidurans* (GCF_000731955), both values below the species delineation threshold of 95%. The result was supported by phylogenomic analysis, which placed the genome as a distinct lineage within the genus *Modicisalibacter* (Figure 4), and confirmed *M. muralis* as the closest relative. The phylogenetic tree also showed a number of *Halomonas* species within the genus *Modicisalibacter*, the result of a taxonomic reclassification with several species being reassigned from the genus *Halomonas* to *Modicisalibacter* (De La Haba et al., 2023).

The *Modicisalibacter muralis* reference genome is fragmented into 21 contigs and has an overall length of 4,138,450 bp. Despite the fragmented nature the overall quality was assessed to be high with 99.85% completeness and 0.89% contamination. In contrast, the genome of *Modicisalibacter* sp. strain 110-V3 is smaller (−255,345 bp) but could be assembled into one continuous circular chromosome (Supplementary Table S1). Compared to the reference genome, *Modicisalibacter* sp. strain 110-V3 had a lower number of predicted coding sequences (−208), but a higher number of rRNA (+2) and tRNA genes (+12). The synteny analysis confirmed that the organism is distinctly different from the reference, with only small, conserved regions. These regions showed local agreement with the reference contigs but also some potential misassemblies or reorganizations (Figure 2B).

An overall comparison between the annotated COG categories revealed that the *M. muralis* had the highest overrepresentation of genes with a function in “Amino acid transport and metabolism” (E), whereas *Modicisalibacter* sp. strain 110-V3 had the highest overrepresentation of genes with a function in “Replication, recombination and repair” (L) (Figure 3C). A more detailed analysis of coding gene annotations revealed 1,170 genes to be unique to *Modicisalibacter* sp. strain 110-V3. Of these 37 had no assigned COG category and 289 were annotated with the category “Function unknown.” The reference, *M. muralis,* had 1,182 specific genes, of which 58 had no category assigned and 287 were assigned to the “Function unknown” category. *Modicisalibacter* sp. strain 110-V3 showed the largest differences to the reference in the categories “Transcription” (K), “Secondary metabolites biosynthesis, transport and catabolism” (Q), “Carbohydrate transport and metabolism” (G), and “Posttranslational modification, protein turnover…” (O). On the other hand, the *M. muralis* had an overrepresentation of specific genes in the categories “Cell motility” (N) and “Intracellular trafficking, secretion, and vesicular transport” (U), and “Cell cycle control, cell division, …” (D) (Figure 3D).

### 3.2 Metabolic pathways

#### 3.2.1 Carbohydrate metabolism

**Figure 5:**
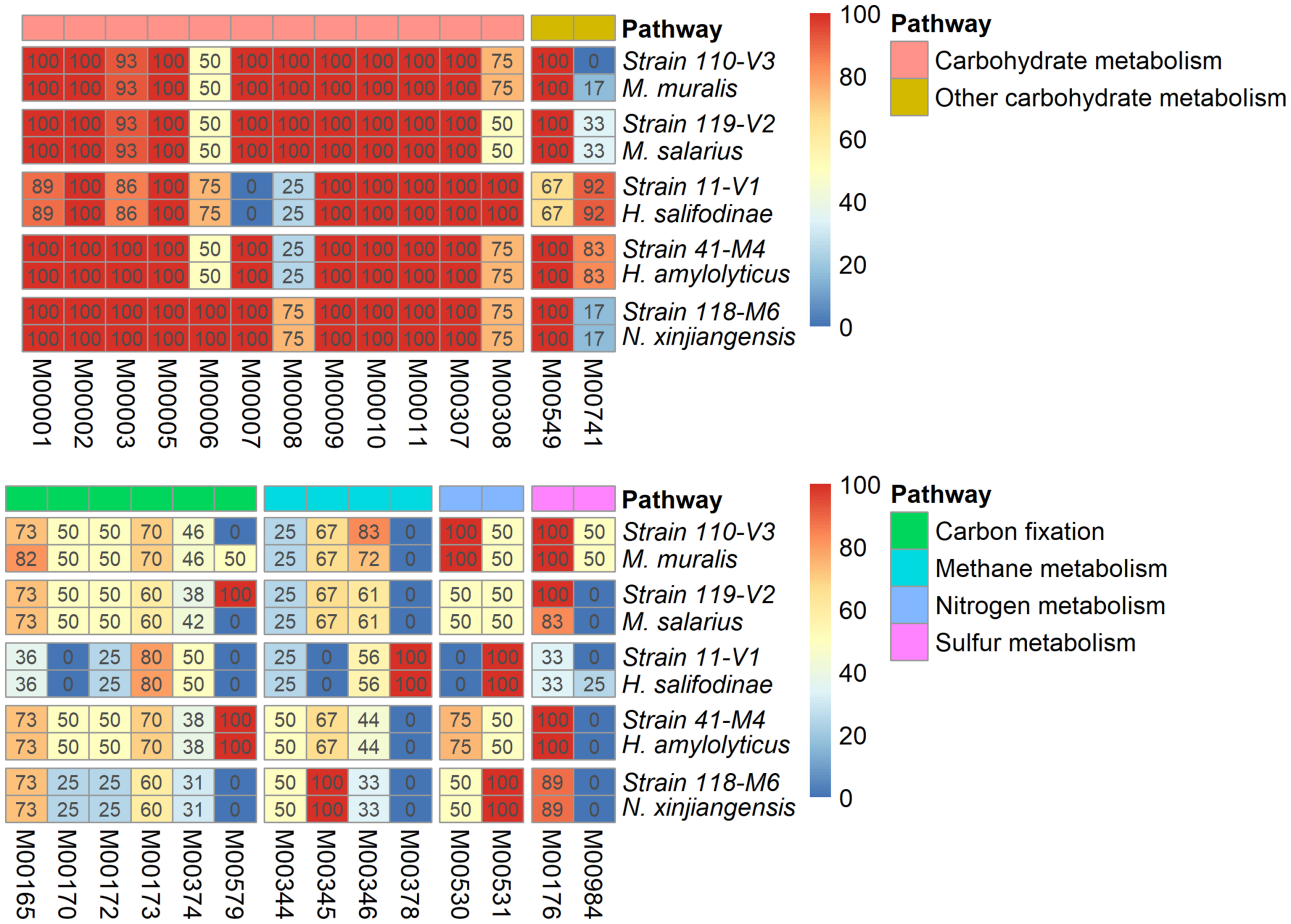
Comparison of selected metabolic pathways between all isolates and their closest relative.

The pathways for glycolysis (M00002), PRPP biosynthesis (M00005), the citrate cycle (M00009, M00010, M00011) and pyruvate oxidation (M00307) were complete in all investigated species and strains (Figure 5). The Embden-Meyerhof pathway (M00001) was complete in all species except for the *Halococcus* strains using an alternative variation. For Gluconeogenesis (M00003) the gene *fbp* (K03841) was replaced by *pfk* (K21071) in both *Marinobacter* species, by *pfkA* (K00850) in both *Modicisalibacter* and by an alternative *pckA* gene variant (K01610) in the *H. salifodinae* strains. The pentose phosphate pathway was only complete in the *N. xinjiangensis* strains. The *Marinobacter* and *Modicisalibacter* species were missing the essential 6-phosphogluconate dehydrogenase (K00033) for the oxidative phase (M00006), and the *H. amylolyticus* strains were missing enzymes for the glucose-6-phosphate dehydrogenase (NADP+/NAD(P)+), while the *H. salifodinae* strains were using an archaeal variation of this step (K19243) but were missing the canonical non-oxidative phase (M00007). The Entner-Doudoroff pathway (M00008) was complete in the *Marinobacter* and *Modicisalibacter* species, while the *H. salifodinae* strains use the semi-phosphorylative Entner-Doudoroff pathway (M00308). The analyzed *N. xinjiangensis* strains were missing the gene *edd* (K01690) in the Entner-Doudroroff pathway (M00008) and *gnaD* (K05308) for the semi-phosphorylative Entner-Doudoroff pathway (M00308), possibly indicating the use of a non-homolog dehydratase. The nucleotide sugar biosynthesis pathway (M00549) was complete in all selected species except for the *H. salifodinae* strains, while these strains showed the highest completion of the Propanoyl-CoA metabolism (M00741) (Figure 5).

#### 3.2.2 Carbon fixation and methane metabolism pathways

*Modicisalibacter* sp. 110-V3 was missing the essential gene *rbcL* (K01601) for the Calvin Cycle (M00165), while this gene was present in the *M. muralis* genome (Supplementary Table S7). None of the genomes contained more than half of the necessary enzymes for the C4-dicarboxylic acid cycle (M00170-M00172), but all showed a good coverage of the reductive citrate cycle (M00173), albeit none encoded a complete enzyme set. The complete phosphate acetyltransferase-acetate kinase pathway (M00579) was found in *Marinobacter* sp. 119-V2 and both *Halobacillus* strains. *M. muralis* was missing *ackA* (K00925) while the *Modicisalibacter* sp. 110-V3 had none of the necessary genes (Figure 5).

Of the methane metabolism pathways for formaldehyde assimilation (M00344, M00345) only *N. xinjiangensis* strains had a complete ribulose monophosphate pathway. The formaldehyde assimilation, serine pathway (M00346), associated with methane metabolism, had a higher completeness in *Modicisalibacter* sp. 110-V3 with the essential AGXT (K00830) gene being detected. The archaea specific F420 biosynthesis pathway (M00378), was complete in *H. salifodinae* strains (Figure 5).

#### 3.2.3 Nitrogen and sulfur metabolism

The pathway for dissimilatory (M00530) nitrogen reduction was complete in both *Marinobacter* species, while completely absent in the *Halococcus* strains, whereas the assimilatory nitrate reduction (M00531) pathway was complete for the *Halococcus* and *Nesterenkonia* strains. The complete assimilatory sulfate reduction pathway (M00176) was found in *Marinobacter* sp. 119-V2, while one gene (*cysJ*) (K00380) was missing in *M. salarius* (Supplementary Table S6).

### 3.3 Metabolic adaptations in relation to saline environments

#### 3.3.1 Osmoregulation

Two major osmoregulation strategies have been described for microorganisms. The “salt-in” strategy involves the active regulation of intracellular ion concentrations through specific ion transport systems, whereas the “salt-out” strategy relies on the accumulation of compatible solutes that stabilize cellular components without disrupting metabolic processes (Oren, 2013; Gunde-Cimerman et al., 2018). This can either happen through synthesis of said solutes or through their uptake from the environment. *Halococcus* sp. strain 11-V1, *Nesterenkonia* sp. strain 118-M6 and *Halobacillus* sp. strain 41-M4 showed no difference to the reference in their osmoregulation potential, but since the osmoregulation strategies and potentials are not well described for all used reference strains, they are included in the description.

With regard to “salt-in” systems, specialized K^+^ uptake and Na^+^ export systems, represented by *trkH* (K03498) and *trkA* (K03499), and the electrochemical potential-driven cation: H^+^ antiporter *yrbG* (K07301) were present in all investigated genomes. The voltage-gated potassium channel *kch* (K10716) was absent from the *Nesterenkonia* strains as well as *Modicisalibacter* sp. 110-V3, in contrast to the reference *M. muralis*. In addition, the *Nesterenkonia* strains, were missing the Na^+^: H^+^ antiporter *nhaP* (K03316) (Supplementary Table S2). A complete multi-subunit Na^+^: H^+^ antiporter complex, *mnhA–G* (K05565–K05571), was only present in the *Halococcus* and *Halobacillus* strains, while the remaining genomes lacked one or more subunits. The chloride channels *clcA/clcB* (K03281) were present only in both *Marinobacter* species and *M. muralis* but not *Modicisalibacter* sp. 110-V3., and the mechanosensitive channel *mscS* (K03442), was found exclusively in both *Marinobacter* species (Supplementary Table S2).

For the “salt-out” system, the sodium/proline symporter *putP* (K11928) and the electrochemical potential-driven glycine betaine transporter *betL* (K05020) were found in all analyzed genomes, whereas the osmoprotectant ABC transport system *opuABCD* (K05845, K05846, K05847) and TC. SSS (K03307) were found in all bacterial genomes but were absent from the archaeal strains. Similarly, genes required for proline biosynthesis (*proABC*), the glycine betaine synthesis pathway from choline (*betA/B*; K00108, K00130) and the complete ectoine biosynthesis pathway (*ectABCD*) were present in all analyzed bacterial genomes but were absent in the archaeal strains (Supplementary Table S2). The transporter *betT/betS* (K02168), involved in the uptake of choline, glycine betaine, and proline betaine, was missing in both *Marinobacter* species and the *Halobacillus* strains. Another BCCT (Betaine/Carnitine/Choline Transporter) family transporter *opuD/betL* (K05020) was found in all genomes except for *M. salaries*, and the trehalose/maltose transport system permease *thuF* (K10237) was only present in the *Marinobacter* sp. strain 119-V2 as well as the *Modicisalibacter* reference *M. muralis*. The trehalose biosynthesis pathway (*otsA/B*; K00697, K01087) was identified in the *Halococcus* and *Nesterenkonia* strains (Supplementary Table S2).

Concerning repair mechanisms *Marinobacter* sp. strain 119-V2, *Modicisalibacter* sp. 110-V3 and *N. xinjiangensis* were missing the gene for the ATP-independent small heat shock protein HSP20 (K13993), instead *Marinobacter* sp. strain 119-V2 contained the DNA repair mechanism OGG1 (K03660), which was not found in the reference (Supplementary Tables S2, S6).

#### 3.3.2 Pigment production

Several KEGG pathways associated with carotenoid biosynthesis, particularly the formation of bacterioruberin, were analyzed for completeness across the genomes. There were few differences to the reference strains, but since these pathways are important in the frame of cultural heritage science and not described for all the analyzed species, our annotation results are listed below.

**Figure 6:**
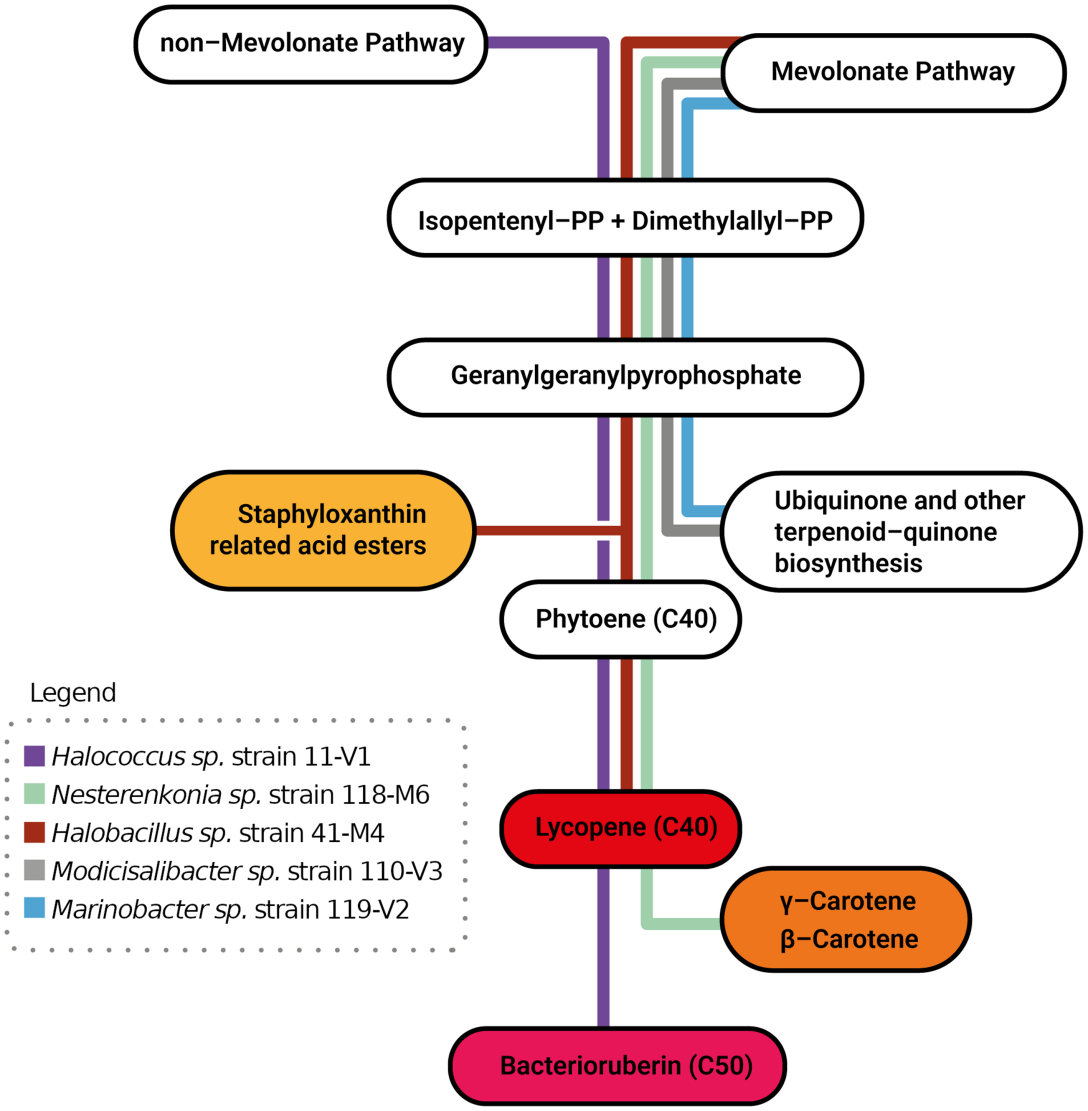
Graphical representation of carotenoid pigment production capabilities of the investigated isolates.

In all bacterial strains and species, a complete set of genes for isoprenoid precursor synthesis via the non-mevalonate pathway (MEP), was detected with minor differences. In both *Marinobacter* and *Modicisalibacter* species, genes for the enzymes IspD and IspF are encoded, whereas the *Nesterenkonia* and *Halobacillus* strains encode the bifunctional enzyme IspDF (Gabrielsen et al., 2004). The analyzed archaeal (*Halococcus*) genomes contain a complete set of genes for isoprenoid precursor formation via the canonical mevalonate pathway (MEV) (Supplementary Table S2; Figure 6).

All genomes contained the genes necessary for the formation of geranylgeranyl pyrophosphate (GGPP), a key intermediate in carotenoid biosynthesis. The *Halococcus* and *Nesterenkonia* strains encoded genes encoding the canonical enzymes Idi (K01823) and IdsA (K13787). In *Modicisalibacter* sp. 110-V3, the gene for *idi* was present, but instead of *idsA*, genes encoding the alternative enzymes IspA (K00795) and GGPS (K13789) were identified. The *Marinobacter* species and the *Halobacillus* strains lacked *idi* but contained genes for an alternative route via *ispH* (K03527) followed alternatively by *ispA* in both *Marinobacter* species, and GGPS in the *Halobacillus* strains (Supplementary Table S2; Figure 6). The gene encoding the phytoene synthase CrtB (K02291), which catalyzes the condensation of two GGPP molecules into the colorless carotenoid phytoene, was present in the *Halococcus* and *Halobacillus* strains. *Nesterenkonia* sp. 118-M6 lacked the *crtB* gene but contained a gene encoding another squalene/phytoene synthase (UniProt ID: A0A7Z0GLE0). In contrast, no enzymes responsible for the conversion of GGPP to phytoene were found in the *Marinobacter* and *Modicisalibacter* species (Supplementary Table S2; Figure 6). Conversion of phytoene to lycopene, a deep red colored carotenoid, via the lycopene synthesis gene *crtI* (K10027) was found in the *Nesterenkonia* and *Halobacillus* strains. In haloarchaea, the lack of a recognizable CrtI-type phytoene desaturase can be functionally compensated by CrtD-like enzymes, acting as bifunctional phytoene desaturases and 3,4-desaturases for bacterioruberin biosynthesis, suggesting that CrtD (which is present in both *Halococcus* strains) can substitute for CrtI (Yatsunami et al., 2024; Yang et al., 2015; Nagar et al., 2024). Pairwise alignment revealed 72.9% amino acid identity over 490 residues between the *Halococcus* sp. 11-V1 protein and the bifunctional CrtI/CrtD-like enzyme C0507 from *Haloarcula japonica*, and 64.6% identity over 486 residues with the related D1086 homolog, with low gap frequencies (0.2–0.4%). Further conversion of the dark red to the reddish-purple C50 carotenoid bacterioruberin requires a series of reactions: two cyclization and modification steps catalyzed by LyeJ (K20616) and CrtD (K20611) and two hydration steps via CruF (K08977). Genes that encode for these three enzymes were only found in the *Halococcus* strains, where they were localized in a genomic gene cluster (*crtB*, *cruF,lyeJ, crtI/crtD*) that was confirmed with AntiSMASH and corresponds in order and orientation to previously described haloarchaea species (Serrano et al., 2022). In the *Nesterenkonia* strains, two distinct lycopene cyclase enzymes were annotated, indicating the potential for conversion of lycopene into the yellow- to orange-colored carotenoids *γ*-carotene and *β*-carotene. The *Halobacillus* strains additionally contained the genes *crtN*, *crtM*, *crtP*, and *crtQ*, associated with the conversion of farnesyl pyrophosphate to yellow staphyloxanthin-related acid esters (Supplementary Table S2; Figure 6).

## 4 Discussion

In this study, the genomes of five organisms that formed part of the microbial community from two investigated historic buildings, were analyzed. Both buildings displayed salt-weathering and salt efflorescence and harbored a microbial community of halophilic and halotolerant microorganisms, determined through targeted 16S rDNA sequencing (Tichy et al., 2023). After sampling the halotolerant/halophilic microorganisms were isolated and enriched in a culture media with high salt concentrations (10 and 20% NaCl) where they exhibited colony coloration ranging from pale yellowish to dark pink. As carotenoid pigments are known to be responsible for pink discolorations associated with salt deposits observed on a range of historic sites (Ettenauer et al., 2014; Tichy et al., 2023; Cojoc et al., 2019; Imperi et al., 2007; Cuzman et al., 2025), the goal was to contribute to the extension of available full genome references for the functional investigation of microorganisms encountered at historic, salt weathered sites. Annotated reference genomes also benefit the validation of future metatranscriptomic data, avoiding low mapping quality and reducing potential misassignment errors. Overall, the whole genome approach adopted in this study supports a cautious but clear refinement of how heritage microbiomes are researched. Amplicon-based surveys have been, and will remain, highly valuable for broad community profiling in cultural heritage research, particularly for capturing overall diversity patterns and enabling cross-study comparisons (Tichy et al., 2023, 2025). At the same time, our data illustrate that amplicon approaches alone may not fully resolve taxonomic relationships or capture strain-level genomic and functional diversity in this niche.

### 4.1 Isolates

The newly assembled isolates are composed of one archaeon, and four bacterial species. Based on the performed ANI and synteny analysis, the archaeal isolate *Halococcus* sp. 11-V1, and the bacterial isolates *Nesterenkonia* sp. 118-M6 and *Halobacillus* sp. 41-M4 likely represent new strains of the species *Halococcus salifodinae, Nesterenkonia xinjiangensis* and *Halobacillus amylolyticus*, respectively. *Marinobacter* sp. 119-V2 and *Modicisalibacter* sp. 110-V3 represent novel species with the closest relative being *Marinobacter salarius* (GCF_002116735, ANI 86.5%) and *Modicisalibacter muralis* (GCF_900102945, ANI 85%).

*Halococcus salifodinae* is an extremely halophilic archaeon capable of growth at high NaCl concentrations (NaCl 15–30% w/v) typical of hypersaline environments (Legat et al., 2013; Denner et al., 1994). It has been found in neutral to slightly alkaline conditions with very low nutrient availability, and frequent desiccation (Denner et al., 1994), which makes it especially successful in conditions found in salt-weathered buildings. The two strains show differences in their nitrogen metabolism, where *Halococcus* sp. strain 11-V1 lacks the nitrate reductase *nirK* (K00368) as well as other genes involved in the conversion of nitrite and ammonia to nitrogen (M00973). In contrast to the reference, the new strain lacks all genes for thiosulfate dependent sulfur oxidation, but contains a 2-phosphosulfolactate phosphatase (*comB*, K05979) (Supplementary Table S3).

The occurrence of closely related strains in geographically distant salt deposits suggests long-term survival in halite environments, partly due to their ability to go into a dormant state (Stan-Lotter et al., 1999). This finding is also supported for the local sampling site, the St. Virgil Chapel, where the genus *Halococcus* was found to be dominant (~⅔ of the community) in a recent study (Tichy et al., 2023), but was already described in a study from 2014 (Ettenauer et al., 2014). Enzymatic studies have demonstrated that extracellular proteases from this organism remain active over a wide range of salt concentrations, (Hong et al., 2023; Hou et al., 2021), and are therefore capable of degrading protein rich historic materials like casein or animal glue. The analyzed strain contained 16 secreted proteases, of which 2 were members of the S08A protein family with the potential to degrade such binders (Supplementary Table S9). Due to its dependence on high salt levels, restoration and desalination treatments can have a strong effect on its abundance as shown by a recent study (Tichy et al., 2025).

*Nesterenkonia xinjiangensis* is a gram-positive, moderately halophilic (8–15% NaCl) and alkalitolerant actinobacterium (Orhan et al., 2023). Many members of the *Nesterenkonia* genus are chemoorganotroph, and although it was not reported for *N. xinjiangensis*, it is supported by the genomic analysis of *Nesterenkonia* sp. 118-M6. Both *N. xinjiangensis* strains have genes for assimilatory nitrate reduction (*nasAB*), enabling it to use nitrogen for growth, as well as genes for nitrite reductase (*nirBD*), which reduces nitrite to ammonia (Supplementary Table S3). The presence of isocitrate lyase and malate synthase in the genome, which enable it to use a glyoxylate shunt, bypassing CO_2_ producing steps of the TCA cycle to conserve carbon for biosynthesis and thereby allowing the organism to grow in nutrient poor environments, were confirmed in the new strain. In contrast to the reference strain, *Nesterenkonia* sp. 118-M6 lacks the ability for glutathione biosynthesis from glutamate (M00118) (Supplementary Table S2).

The species could not be detected through 16S rDNA analysis (Tichy et al., 2023) of the sampled historic sites but was one of the organisms that emerged after the walls were treated with desalination poultices (Tichy et al., 2025). Since the samples from which the presented isolates were cultured, were taken before the treatment, the organism was already present in the native wall community under the detection limit and got enriched through the desalination treatment. This example shows that restoration activities can have unforeseen consequences with regard to the microbial colonization of the surface or substrate, and that it is important to evaluate such microbial interactions in the frame of heritage science.

*Halobacillus amylolyticus* is a moderately halophilic, gram-positive bacterium (Kim et al., 2023a) and has been reported to grow under salt concentrations of up to 20% NaCl (Ripka et al., 2006). *Halobacillus* is a genus well known for its broad salt tolerance and environmental robustness with the ability to form endospores (Amoozegar et al., 2003; Piñar et al., 2001). Some *Halobacillus* species are able to utilize starch and produce extracellular enzymes (e.g., amylases), which may be beneficial under carbon limitation (Ripka et al., 2006; Li and Hui-Ying, 2011). Alpha-amylase was annotated in *Halobacillus* sp. 41-M4, indicating its capability to convert starch to maltose and dextrin, but further experimental characterization of the strain is needed to confirm this potential. The strain also contains a cytochrome bd ubiquinol oxidase (M00153), that is missing from the reference (Supplementary Table S2). These oxidases are favored under microaerophilic or stress conditions, supporting respiration when O₂ is low and often contributing to resistance against nitrosative and oxidative stress (Borisov et al., 2021).

Species of the genus *Halobacillus* were found in salt-weathered historic buildings in Austria before (Ripka et al., 2006) and were also detected in low abundances in the 16S rDNA analysis of the sampling sites (Tichy et al., 2023). It has to be stressed that the 16S rDNA based identification of genera and species within the order *Bacillales* can be challenging due to the low sequence variability within the group, and misassignments are common (Nunes Ramos et al., 2025).

*Marinobacter salarius* is a gram-negative, moderately halophilic bacterium capable of growth at salinities up to 20% NaCl (Ng et al., 2014). In contrast to the reference, *Marinobacter* sp. 119-V2 contains the cytochrome o ubiquinol oxidase (M00417), a proton-pumping oxidase that can maximize energy conservation from limited organic substrates on salt-weathered surfaces where oxygen is usually non-limiting; and the phosphate acetyltransferase-acetate kinase pathway (M00579), which functions as a central ATP-generating branch to dispose of acetyl-CoA (Wolfe, 2005). Additionally, *Marinobacter* sp. 119-V2 lacks the capability to degrade benzoate (M00551), compared to the reference (Supplementary Table S2), which may be an adaptation to salt-weathered mineral surfaces, where aromatics are present only at low background levels. In contrast to the reference, the analyzed isolate did not contain a plasmid.

Species of the genus *Marinobacter* were detected at the St. Virgil Chapel in moderate abundances (6.2–16.7% rel. abundance) (Tichy et al., 2023). The presence of *Marinobacter* sp. 119-V2 at this site confirms the capability of *Marinobacter* species to successfully and persistently colonize hypersaline environments. But a recent study at the investigated sites also showed that desalination treatment was able to reduce the abundance of this species up to a point where it could not be detected anymore (Tichy et al., 2025).

*Modicisalibacter muralis* (formerly *Halomonas muralis*, De La Haba et al., 2023), a heterotrophic, gram-negative bacterium with optimal growth conditions at up to 10% NaCl was already isolated from microbial biofilms colonizing walls and murals in a historic building (Heyrman et al., 2002). Other species of the genus *Modicisalibacter* (formerly *Halomonas*) were found in saline and hypersaline marine and terrestrial environments and are reported to be highly halotolerant (up to 30% NaCl) (Kim et al., 2023b). Many *Modicisalibacter* species produce exopolysaccharides (EPS) as a protective barrier against salt stress and to retain water and nutrients (Athmika et al., 2021). In the assembled isolate various ABC-transporters, including genes for the glycerol-3-phosphate transporter (*ugpAEC*), the L-arabinose transporter (*araHGF*), and the phospholipid transporter (*mlaFED*), as well as *lptB* were detected. The strain lacked the for *Halomonas malpeensis* described *kps* genes (Athmika et al., 2021), but encodes several other genes involved in biofilm formation (*wzc*, *wza*, *wbpM*, *wbpP*, *yqjZ*) (Supplementary Table S7). There are several pathways that show differences to the reference (Supplementary Table S2), unfortunately they do not form a clear adaptive genotype, and would need more detailed and experimentally supported research to elucidate.

*Halomonas* species were detected at the St. Virgil Chapel with strong local abundance differences (0.7–49.7% rel. abundance) (Tichy et al., 2023).

### 4.2 Metabolic adaptations in relation to saline environments

#### 4.2.1 Osmoregulation

Genome annotation of the five halophilic/halotolerant isolates revealed a diverse repertoire of genes associated with adaptation to high salt, including ion transport systems, Na^+^: H^+^ antiporters, and numerous transport and biosynthetic pathways for compatible solutes. These features fit well into the two canonical osmoadaptive strategies described for halotolerant bacteria and archaea: a “salt-in” strategy based on intracellular accumulation of inorganic ions, and a “salt-out” strategy relying on synthesis or uptake of organic osmolytes such as ectoine, glycine betaine, and proline (Oren, 2013; Gunde-Cimerman et al., 2018).

Consistent with previous reports (Youssef et al., 2014) on *H. salifodinae*, *Halococcus* sp. 11-V1 lacks several compatible-solute biosynthesis and uptake pathways (e.g., ectoine synthesis and *opuABCD* gene cluster) but has the capacity for trehalose synthesis (genes *otsA/B*) and a limited number of osmoprotectant transporters like the glycine betaine transporter BetL and the sodium/proline symporter PutP. The comparatively rich complement of ion transport and Na^+^: H^+^ antiporter systems in this *Halococcus* still indicates a strong reliance on the salt-in strategy (Supplementary Table S2). Although, it is widely accepted that many haloarchaea primarily rely on salt-in mechanisms, research on *Halococcus hamelinensis* has demonstrated that certain species within this genus exhibit a preference for the accumulation of compatible solutes such as glycine betaine, trehalose, and glutamate, rather than intracellular K^+^ (Goh et al., 2011). This highlights the diverse salt adaptation strategies in the genus *Halococcus*.

By contrast, the widespread presence of genes for ectoine (*ectABCD*), proline (*proABC*) and glycine betaine synthesis from choline (*betAB*) in the four bacterial genomes, as well as multiple uptake systems (e.g., *betL*, *putP*, *opuABCD* and *TC. SSS*) show greater genetic potential for salt-out osmoadaptation, which aligns well with prior work on the analyzed genera (Fernandez-Linares et al., 1996; Kim et al., 2017; Marsh et al., 2021). *Nesterenkonia* species such as *N. xinjiangensis* are industrially exploited for ectoine production, demonstrating robust ectoine biosynthesis under high-salt conditions and confirming the central role of compatible solutes in this genus (Orhan et al., 2023). Our analysis showed that both *N. xinjiangensis* strains also have the potential for trehalose synthesis, in contrast to the other investigated bacterial species. *Marinobacter* sp. 119-V2 encoded the osmoprotectant transporter BetL, and the trehalose/maltose transport system permease protein ThuF, which were both not present in the reference, potentially indicating an adaptation to the saline environment (Supplementary Table S2). *Modicisalibacter* sp. 110-V3 lacked three transporters that the reference genome contained, the chloride channel protein ClcA, the voltage-gated potassium channel protein Kch, and the trehalose/maltose transport system permease protein ThuF. Instead of Kch *Modicisalibacter* sp. 110-V3 may use the Trk/Ktr system potassium uptake protein (K03498), which was not annotated in the reference (Supplementary Table S2). For the other missing transporters no specific replacement could be found, suggesting either no need for this specific chloride and trehalose/maltose transporter or the usage of non-specific transporters.

#### 4.2.2 Pigment production

Pink biofilms are commonly associated with hypersaline environments (Villa et al., 2022), where elevated salinity causes cellular desiccation, membrane stress, and the formation of reactive oxygen species (ROS) (Lebre et al., 2017). Such harsh conditions favor carotenoid-producing microorganisms that utilize these compounds in the mitigation of oxidative stress (Squillaci et al., 2017), the protection against high UV radiation (Takano et al., 2005), and the stabilization of membranes (Flegler and Lipski, 2021). The genomic analyses of the five isolated strains indicate that they employ distinct pigment biosynthesis strategies (Figure 6).

The *Halococcus* sp. strain 11-V1 genome encodes the complete mevalonate pathway (MEV), GGPP synthase, and the haloarchaeal bacterioruberin biosynthesis module (*crtB, cruF lyeJ,* and *crtI/crtD*), mirroring the experimentally validated C50 bacterioruberin pathway of *Haloarcula japonica* (Yang et al., 2015) (Figure 6). The genomic evidence is consistent with the deep pink to reddish pigmentation observed in culture and aligns with previous descriptions of *H. salifodinae* (Denner et al., 1994). It also identifies this strain as the source of the bacterioruberin-associated Raman peaks of the salt-weathered surface of the St. Virgil Chapel from which strain 11-V1 was isolated (Tichy et al., 2023). To the best of our knowledge, this is the first study to describe the complete bacterioruberin biosynthesis gene complement in a *H. salifodinae* strain, extending previous reports of its pink pigmentation to a genomic basis for bacterioruberin production.

The genome of *Nesterenkonia* sp. strain 118-M6 encodes the complete non-mevalonate (MEP) pathway together with the enzymes required for lycopene formation and its conversion into C40 carotenoids such as *γ*-carotene and *β*-carotene (Figure 6). Since lycopene is a deeply red carotenoid, whereas downstream cyclized derivatives are typically yellow to orange, the observed orange-pink colony coloration is most plausibly explained by the varying ratios of these pigments.

In *Halobacillus* sp. strain 41-M4, the presence of the *crtB* and *crtI* genes together with *crtM*, *crtN*, *crtP* and *crtQ* is consistent with a C30 staphyloxanthin-like pathway that has been characterized in *H. halophilus*, where CrtM and CrtN form yellow C30 carotenoid backbones that are further modified to orange staphyloxanthin esters with demonstrated protective roles against oxidative stress (Köcher et al., 2009) (Figure 6). Importantly, this finding extends the occurrence of a staphyloxanthin-like carotenoid biosynthetic repertoire to *Halobacillus amylolyticus* strain 41-M4, indicating broader carotenoid diversity within the genus than previously known. The coexistence of C40 and C30 pathways in *Halobacillus* therefore likely yields mixed carotenoid pools, and differences in the relative abundance of red lycopene-derived pigments versus yellow C30 esters can account for the observed subtle shifts in colony hue among the carotenoid-producing bacteria. Both the *N. xinjiangensis* strain 118-M6 and *H. amylolyticus* strain 41-M4 can be major contributors to discoloration seen in cultural heritage sites when present.

The *Marinobacter* sp. strain 119-V2 and *Modicisalibacter* sp. strain 110-V3 encode the full MEP pathway and GGPP synthase but lack *crtB*/*crtI* homologs, indicating that GGPP is produced but its further conversion in a canonical C40 carotenoid pathway is not supported (Figure 6). In bacteria, GGPP and related prenyl diphosphates are central branch points not only for carotenoids but also for the synthesis of glycosyl carrier lipids and the polyprenyl side chains of respiratory quinones (Kawamukai, 2018; Manat et al., 2014). Members of the family *Halomonadaceae*, including *Modicisalibacter*, are aerobic halophiles that rely on respiratory quinones as core components of their electron transport metabolism, with *Modicisalibacter xianhensis* JCM 14849 reported to produce ubiquinone Q-9 as its predominant quinone (De La Haba et al., 2023; Zhao et al., 2012). On the other hand, GGPP may be diverted into non-canonical carotenoid pathways even when canonical *crtB-like* genes are not readily identifiable (Cheng, 2006; Klassen, 2010), and several *Marinobacter* isolates from hypersaline and marine environments have been reported to form yellow to orange-pigmented colonies, consistent with carotenoid or related isoprenoid pigment production (Sánchez-Porro et al., 2024). Therefore, the pale pink coloration of *Marinobacter* sp. 119-V2 may indicate low-level production of carotenoid or carotenoid-like pigments, whereas the beige-to-yellow pigmentation of *Modicisalibacter* sp. 110-V3 may reflect the presence of low-abundance or structurally atypical carotenoid pigments and could additionally include contributions from flavin-derived chromophores such as riboflavin derivatives. Both isolates encode the necessary genes for riboflavin synthesis (*ribABDEH*) (Supplementary Table S3).

## 5 Conclusion

In summary, this study presents *de novo* genome assemblies and functional genomic analyses of one halophilic and four halotolerant, pigment-producing microorganisms isolated from highly saline cultural heritage sites. Methodologically, this work demonstrates the value of combining classical microbiological isolation with modern genomic analysis in the study of cultural heritage microbiomes. Cultivation-based approaches allow direct observation of phenotypes, such as pigmentation and salt tolerance, while genome sequencing provides the means to link these traits to underlying genetic determinants.

By elucidating the genomic features associated with pigment production, salt tolerance, and environmental adaptation, the work enhances our understanding of the functional roles played by microorganisms in heritage contexts. The presence of multiple, sometimes overlapping, salt tolerance strategies within individual genomes shows the evolutionary importance of osmoadaptation in heritage-associated halophiles and suggests a high degree of resilience to environmental fluctuations. The discovery of two novel species further highlights the largely unexplored microbial diversity associated with cultural heritage environments and shows that anthropogenically influenced sites represent unique environments that lead to specific evolutionary adaptation and the emergence of specialized lineages with distinct functional capacities. Finally, the genomic characterization of new species expands the foundational knowledge needed to assess potential risks and benefits associated with microbial activity on heritage materials.

Collectively, these findings contribute to the growing body of research on the microbiomes of art and their importance in preserving cultural heritage, emphasizing the need for genomic approaches to address current challenges and to inform future conservation strategies. From a conservation perspective, the findings of this study contribute to a more nuanced understanding of microbial colonization of cultural heritage sites. Pigment-producing halophiles are often viewed primarily as agents of aesthetic damage, yet their persistence is underpinned by sophisticated physiological and genomic adaptations. Recognizing these adaptations is essential for designing effective mitigation strategies. For example, if not carefully designed, interventions aimed at altering surface salinity or moisture levels may inadvertently select for microorganisms that pose an even greater risk to the cultural heritage site or object or in the worst case represent a health risk. Genome-informed insights into osmotic stress responses and metabolic flexibility can help predict how microbial communities might respond to conservation treatments, environmental change, or shifts in site usage.

## Data Availability

The datasets presented in this study can be found in online repositories. This Whole Genome Shotgun project has been deposited at DDBJ/ENA/GenBank under the accession JBZCVU000000000-JBZCVY000000000, with the bioproject number PRJNA1452464. The version described in this paper is version JBZCVU010000000-JBZCVY010000000.
