# Supplementary material for: Expanding genomic resources for heritage science: characterization of selected microbial isolates from salt-weathered historic sites
Source: Front Microbiol. 2026 Jul 2;17:1854786. doi: 10.3389/fmicb.2026.1854786 (PMC13373138; doi:10.3389/fmicb.2026.1854786)
Supplement: Supplementary file 3 [file Table_3.DOCX]

Supplementary Material


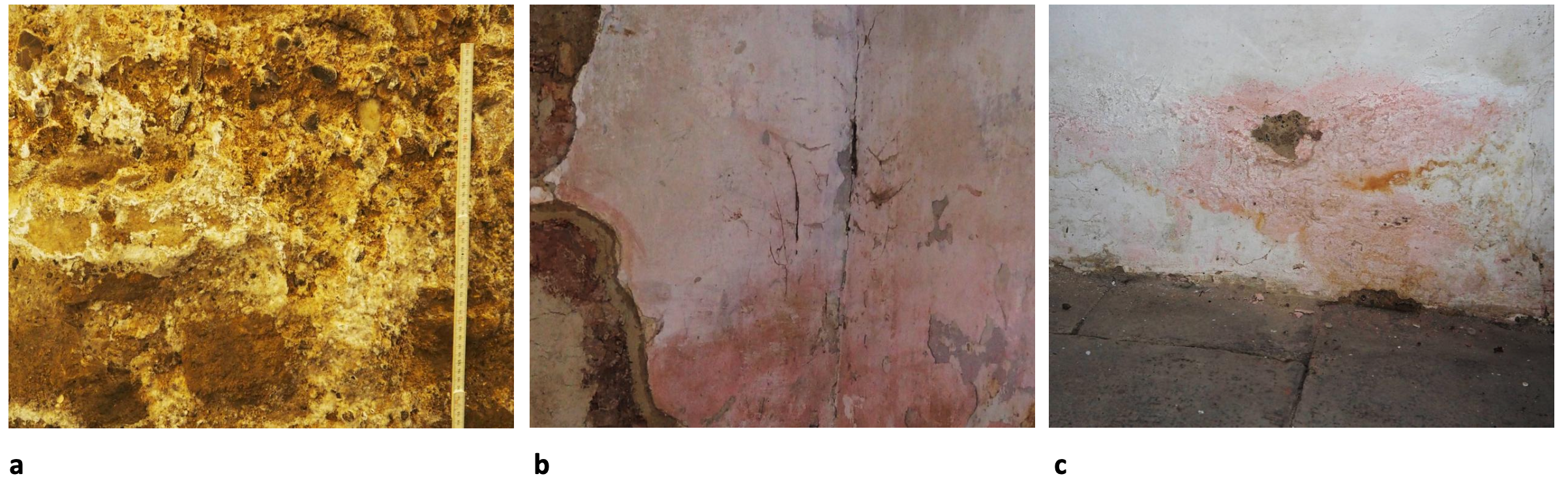


**Supplementary Figure 1.** Pink biofilms on salt-weathered surface with the chosen sampling locations: St. Virgil Chapel (**a**), the charterhouse Mauerbach room 1 (**b**) and room 2 (**c**)


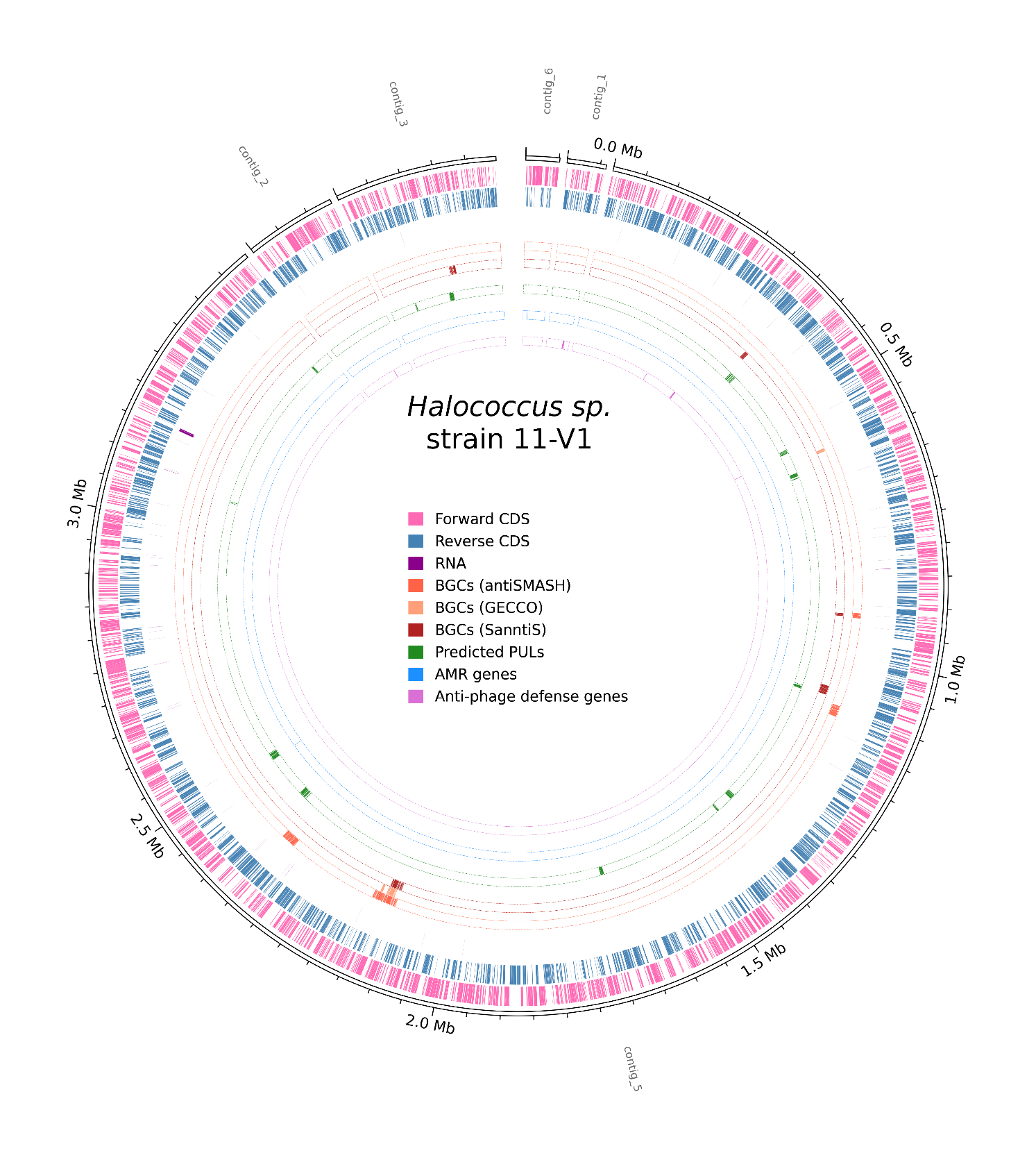


**Supplementary Figure 2.** Depiction (CircosPlot) of the assembled genome and the distribution of annotated genomic features of *Halococcus sp.* strain 11-V1


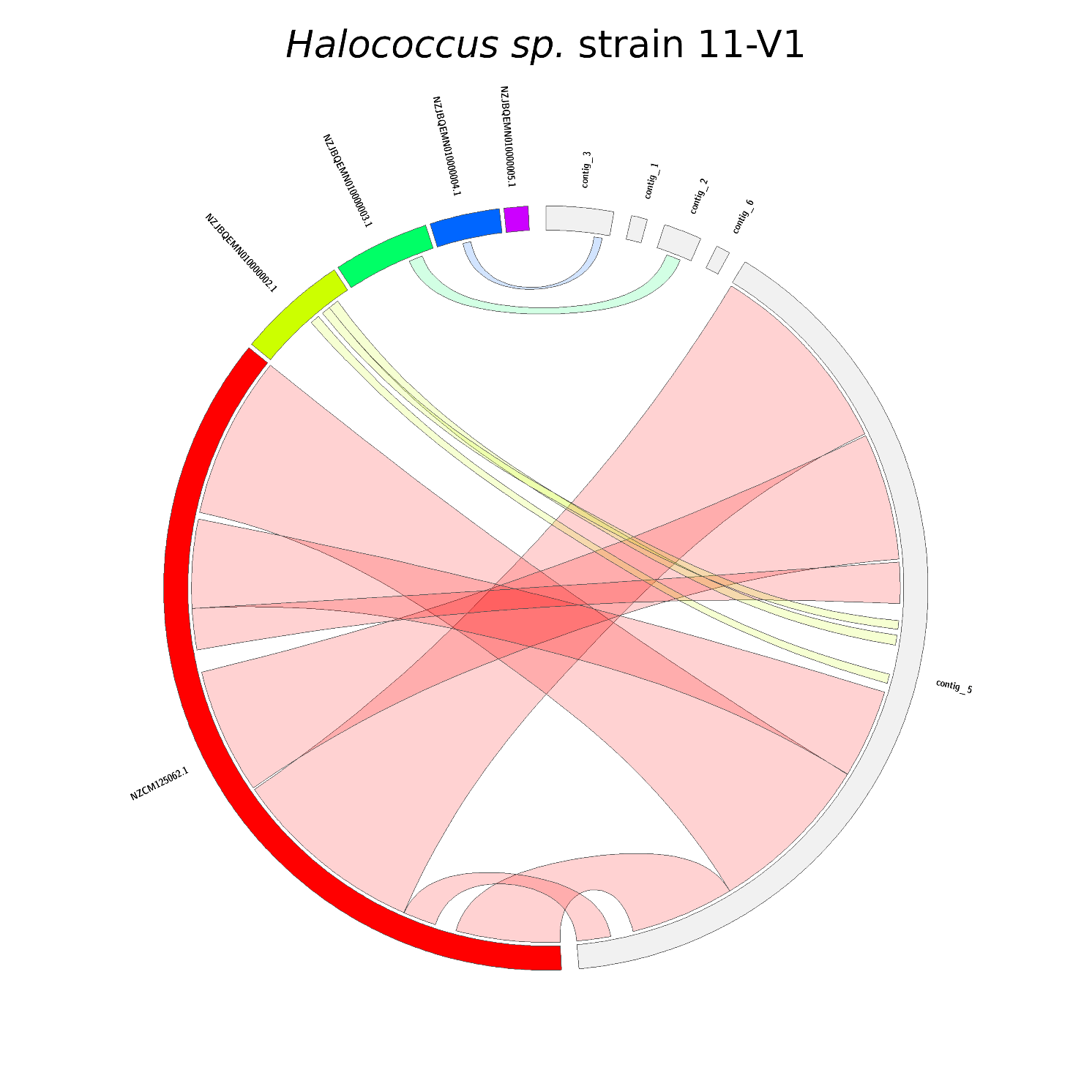
**Supplementary Figure 3.** Synteny plot of *Halococcus sp.* strain 11-V1 and *Halococcus salifodinae*


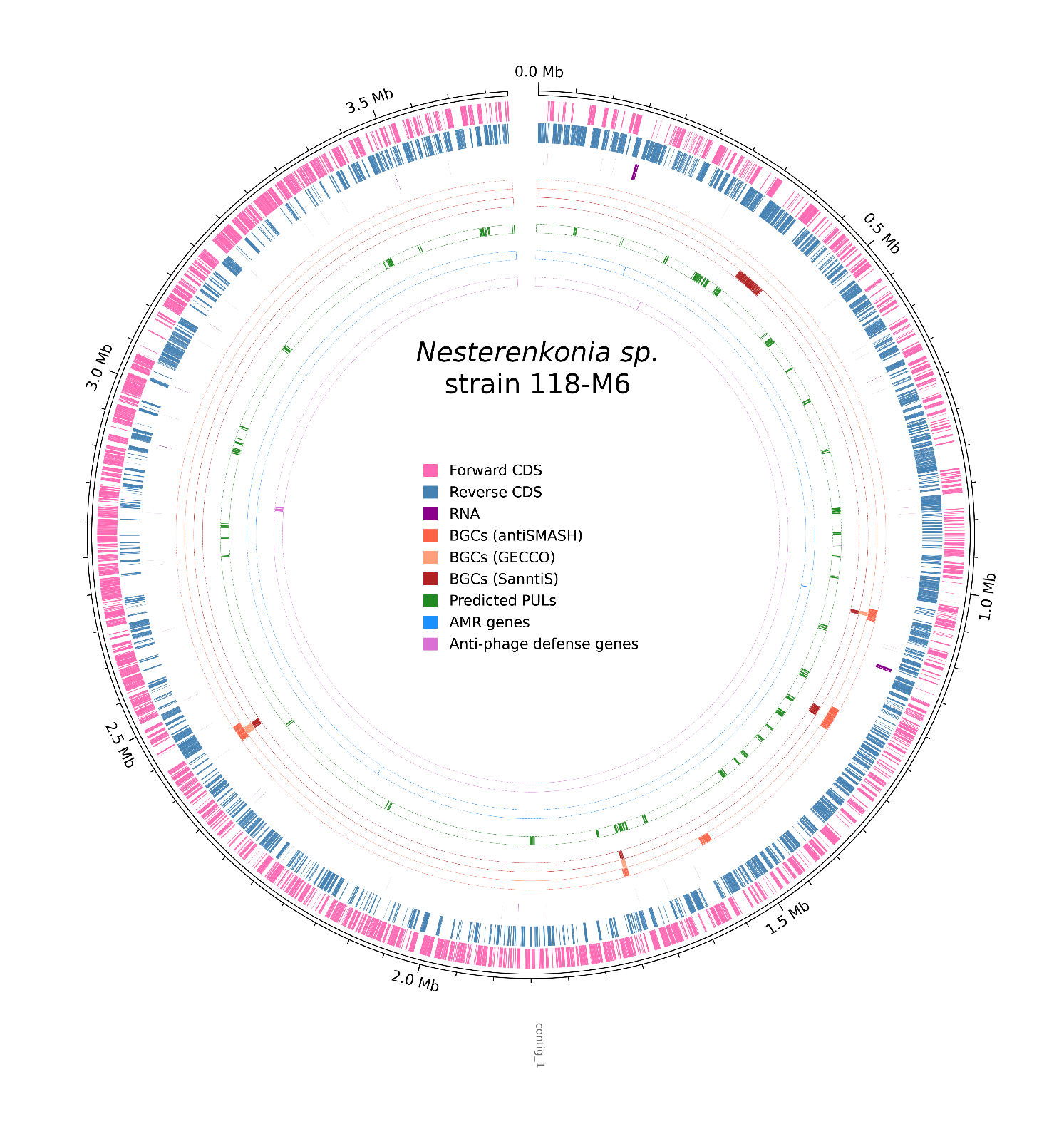


**Supplementary Figure 4.** Depiction (CircosPlot) of the assembled genome and the distribution of annotated genomic features of *Nesterenkonia sp.* strain 118-M6


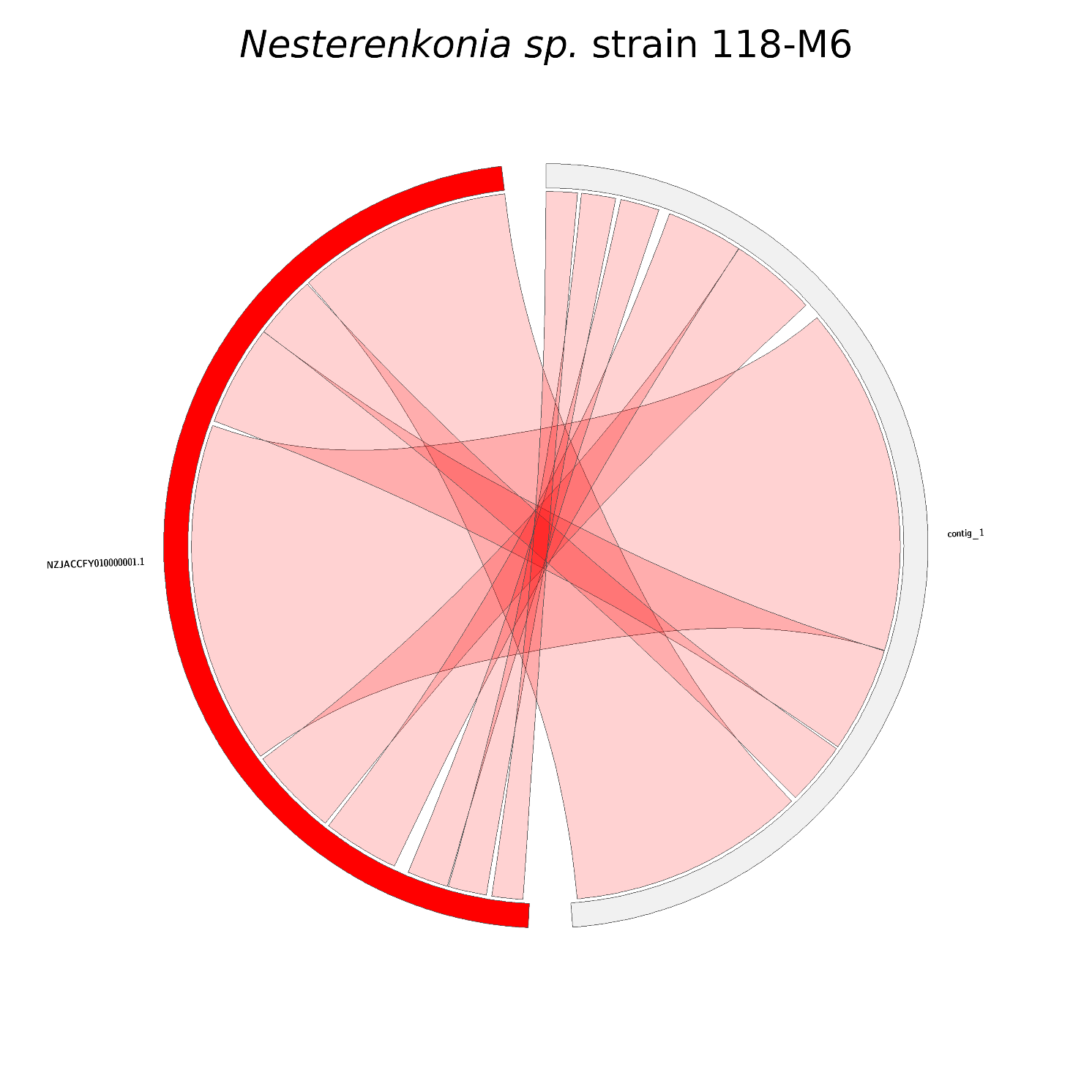


**Supplementary Figure 5.** Synteny plot of *Nesterenkonia sp.* strain 118-M6 and *Nesterenkonia xinjiangensis*


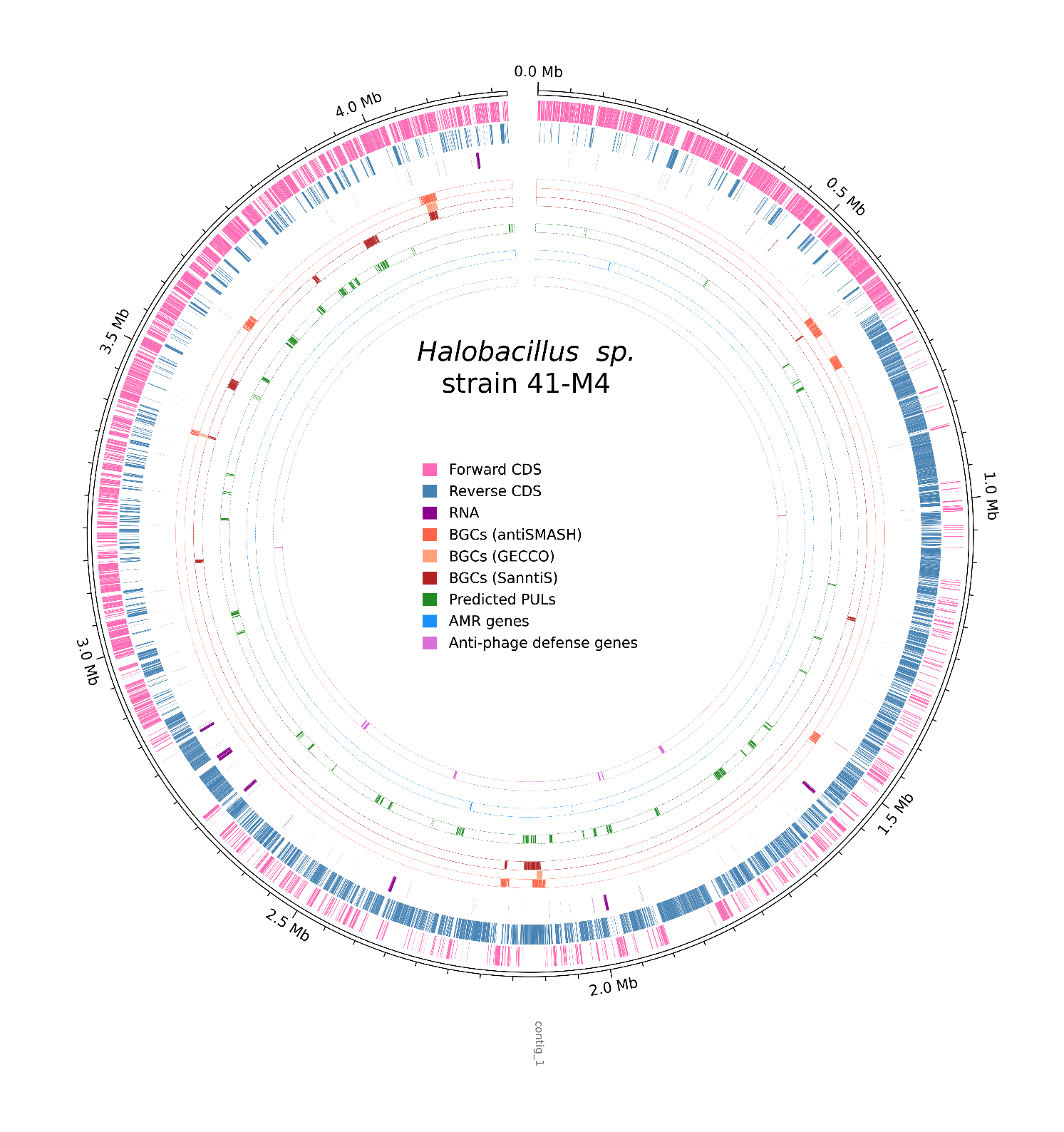


**Supplementary Figure 6.** Depiction (CircosPlot) of the assembled genome and the distribution of annotated genomic features of *Halobacillus sp*. strain 41-M4

*
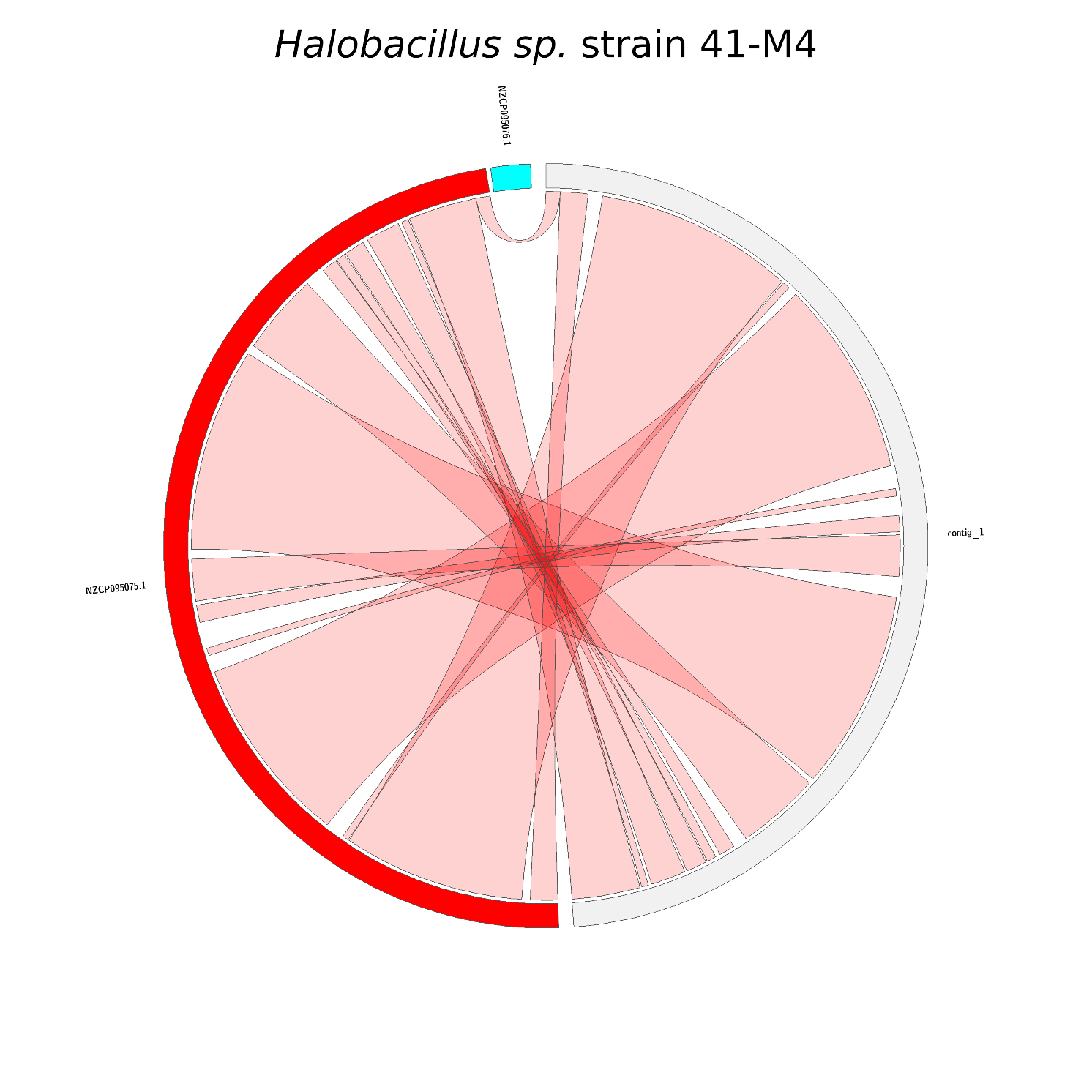
*

**Supplementary Figure 7.** Synteny plot of *Halobacillus sp*. strain 41-M4 and *Halobacillus amylolyticus*


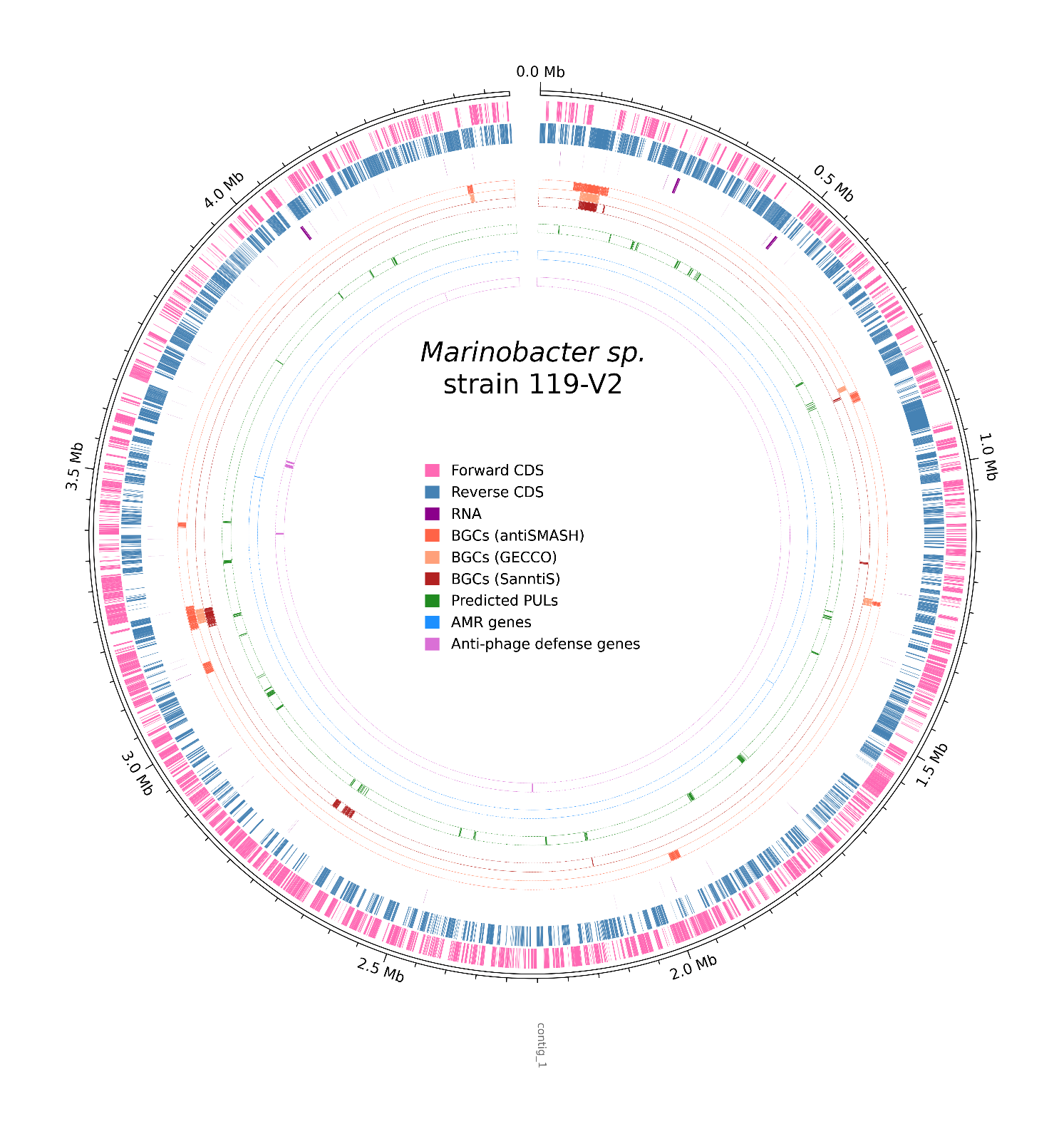


**Supplementary Figure 8.** Depiction (CircosPlot) of the assembled genome and the distribution of annotated genomic features of *Marinobacter sp.* strain 119-V2


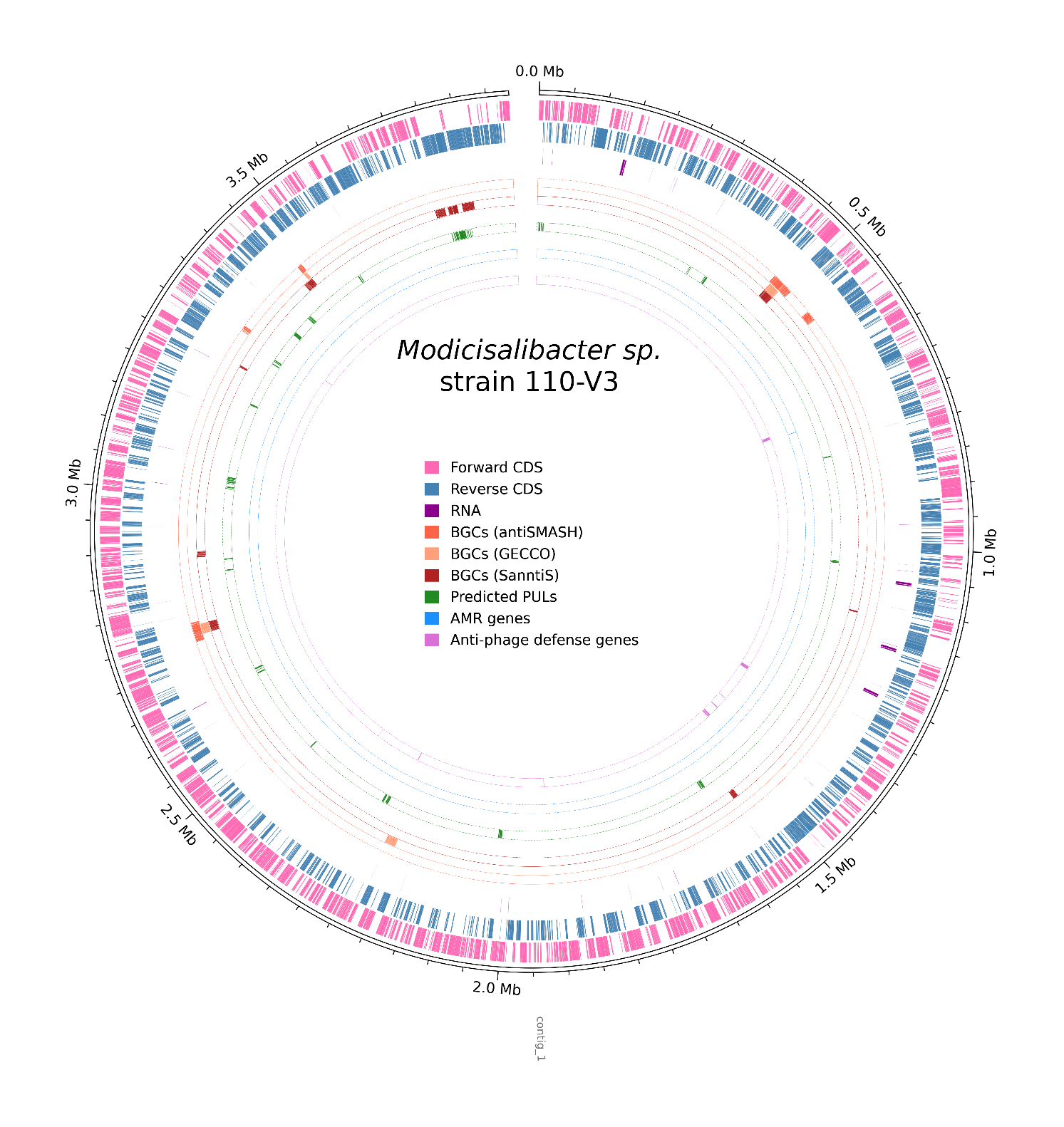


**Supplementary Figure 9.** Depiction (CircosPlot) of the assembled genome and the distribution of annotated genomic features of *Modicisalibacter sp.* strain 110-V3
